# Identification and Functional Analysis of Light-Responsive Unique Genes and Gene Family Members in Rice

**DOI:** 10.1371/journal.pgen.1000164

**Published:** 2008-08-22

**Authors:** Ki-Hong Jung, Jinwon Lee, Chris Dardick, Young-Su Seo, Peijian Cao, Patrick Canlas, Jirapa Phetsom, Xia Xu, Shu Ouyang, Kyungsook An, Yun-Ja Cho, Geun-Cheol Lee, Yoosook Lee, Gynheung An, Pamela C. Ronald

**Affiliations:** 1 Department of Plant Pathology, University of California Davis, Davis, California, United States of America; 2 Functional Genomic Center, Pohang University of Science and Technology, Pohang, Republic of Korea; 3 The Appalachian Fruit Research Station, USDA-ARS, Kearneysville, West Virginia, United States of America; 4 J. Craig Venter Institute, Rockville, Maryland, United States of America; 5 College of Business Administration, Konkuk University, Gwangjin-gu, Seoul, Republic of Korea; 6 School of Veterinary Medicine, Department of Pathology, Immunology and Microbiology, University of California Davis, Davis, California, United States of America; Stanford University School of Medicine, United States of America

## Abstract

Functional redundancy limits detailed analysis of genes in many organisms. Here, we report a method to efficiently overcome this obstacle by combining gene expression data with analysis of gene-indexed mutants. Using a rice NSF45K oligo-microarray to compare 2-week-old light- and dark-grown rice leaf tissue, we identified 365 genes that showed significant 8-fold or greater induction in the light relative to dark conditions. We then screened collections of rice T-DNA insertional mutants to identify rice lines with mutations in the strongly light-induced genes. From this analysis, we identified 74 different lines comprising two independent mutant lines for each of 37 light-induced genes. This list was further refined by mining gene expression data to exclude genes that had potential functional redundancy due to co-expressed family members (12 genes) and genes that had inconsistent light responses across other publicly available microarray datasets (five genes). We next characterized the phenotypes of rice lines carrying mutations in ten of the remaining candidate genes and then carried out co-expression analysis associated with these genes. This analysis effectively provided candidate functions for two genes of previously unknown function and for one gene not directly linked to the tested biochemical pathways. These data demonstrate the efficiency of combining gene family-based expression profiles with analyses of insertional mutants to identify novel genes and their functions, even among members of multi-gene families.

## Author Summary

Rice, a model monocot, is the first crop plant to have its entire genome sequenced. Although genome-wide transcriptome analysis tools and genome-wide, gene-indexed mutant collections have been generated for rice, the functions of only a handful of rice genes have been revealed thus far. Functional genomics approaches to studying crop plants like rice are much more labor-intensive and difficult in terms of maintaining the plants than when studying Arabidopsis, a model dicot. Here, we describe an efficient method for dissecting gene function in rice and other crop plants. We identified light response-related phenotypes for ten genes, the functions for which were previously unknown in rice. We also carried out co-expression analysis of 72 genes involved in specific biochemical pathways connected in lines carrying mutations in these ten genes. This analysis led to the identification of a novel set of genes likely involved in these pathways. The rapid progress of functional genomics in crops will significantly contribute to overcoming a food crisis in the near future.

## Introduction

Gene inactivation by the insertion of T-DNA is a common tool used for functional studies of genes in model plants such as Arabidopsis or rice. T-DNA insertional mutants have been generated for virtually all of the annotated genes in *Arabidopsis thaliana*. Recently, researchers working with Arabidopsis have combined the use of microarray technology with screens of T-DNA insertional mutant libraries to identify and characterize genes of unknown function. This strategy has successfully been used to characterize genes related to the formation of the secondary cell wall, those differentially regulated in phytochrome-mediated light signals, and those involved in host responses to pathogens [1],[2],[3]. These studies did not address the issue of multi-gene families, which limits functional analysis of genes in many plant species.

Rice is a staple crop for more than half of the world's population and a model for other cereal crops. Therefore, studies of the rice genome are expected to help elucidate the function of genes in other major cereal crops, all of which have much larger genomes than rice. So far the functions of only a handful of rice genes have been characterized. The rice research community has recently generated a large collection of rice insertional mutant lines and thus far 27,551 gene loci with knockout mutations have been collected and 172,500 flanking sequences tagging those mutations have been sequenced (http://signal.salk.edu/RiceGE/RiceGE_Data_Source.html) [4]. Four microarray platforms covering nearly the entire rice transcriptome have also been developed [4],[5],[6],[7]. Accordingly, studies similar to those carried out in Arabidopsis, combining microarray-derived expression data with reverse genetics to address gene function, can now be carried out in rice.

Plants are continuously exposed to biotic and abiotic factors, light being one of the most important. In addition to providing the source of energy, light is also involved in regulating growth and development throughout the plant life cycle [8]. Genome-wide gene expression profiles of various light responses can help reveal the complicated physiological networks with which plants adapt to environmental changes. Toward that end, plant researchers have carried out microarray experiments with various plant species in efforts to identify light-regulated genes [9],[10],[11].

One of the major challenges facing scientists in the field of functional genomics, even those working with relatively simple model organisms like Arabidopsis, is the prevalence of gene families [12],[13]. Such family members often encode redundant functions [14],[15]. Because of the presence of gene families with functional redundancy, it is often the case that a gene, when mutated, will display no detectable phenotype. For example, an Arabidopsis line carrying a T-DNA insertion in a gene encoding a protein with a putative R3H domain, *At5g05100*, hypothesized to be of importance in seed organ development, was reported to have no observable mutant phenotype [13]. Because *Arabidopsis* also has three genes, *At2g40960*, *At3g10770*, and *At3g56680*, the lack of a mutant phenotype in this line is probably due to one or more of these genes encoding a function redundant of *At5g05100* [13]. Efforts to overcome such functional redundancy have mostly focused on generating mutants that disrupt more than one gene family member simultaneously [16],[17]. For example, RNA interference (RNAi) technology has also been used to simultaneously silence multiple members of a gene family [18].

Rice researchers encounter even larger gene families, more difficulty in scaling up experiments and greater time constraints associated with rice's longer life cycle than do researchers working with Arabidopsis. For example, whereas Arabidopsis has more than 600 genes in its receptor-like kinase (RLK) family, 80 in the bric-a-brac/tramtrack/broad (BTB) protein family, and 37 genes in the GARS family, rice has more than 1131, 149, and 52 members in those families, respectively [19],[20],[21]. Accordingly, a strategy for overcoming these difficulties and accelerating functional genomics analysis is especially helpful in crops like rice.

Interestingly, the presence of multiple gene family members does not always mask phenotypic changes in gene “knockout” mutants. For example, 43.9% of the Arabidopsis seedling-lethal mutants and 45.2% of the Arabidopsis embryo-defective mutants phenotypically identified in two Arabidopsis studies carried defects in genes that were members of gene families [22],[23]. These results suggest that all members of gene families are not necessarily functionally equivalent and individuals among them may play predominant roles [24].

Here we report the use of a microarray that covers nearly the entire rice transcriptome, the National Science Foundation-supported 45K microarray (NSF45K, http://www.ricearray.org/), to identify light-responsive genes in rice and subsequent functional analysis of a subset of those genes through screening gene-indexed mutant lines of rice. We describe the advantages of utilizing candidate genes derived from rice gene expression profiles to screen rice insertional mutant collections and discuss the biological significance of our findings.

## Results/Discussion

### Light-Responsive Genes Were Identified in Rice from Four Different Genetic Backgrounds

We performed expression-profiling experiments with a newly-developed NSF45K microarray (http://www.ricearray.org/) using two weeks-old rice leaf tissues harvested from plants grown under light and dark conditions (See Materials and Methods). Because genetic backgrounds can affect expression profiles [25] and we wanted to select light-responsive genes that behaved similarly in different genetic backgrounds, identical experiments were carried out with four different rice varieties: Kitaake, Nipponbare, Tapei309 and IR24.

We used the LMGene Package developed by Rocke (2004) to identify 10361, 4962, 1933, and 453 genes differentially expressed in response to light versus dark treatment at FDR p-values of 0.01, 10^−4^, 10^−6^, and 10^−8^, respectively (Table S1). To assess the difference in gene expression profiles among the varieties, we calculated correlation coefficients. Correlation coefficient values were 0.89–0.92 between the three japonica varieties (i.e. Kitaake, Nipponbare, and Tapei309) whereas correlation coefficients values between subspecies (i.e. japonica and indica) were 0.80–0.82. This result indicates that differences in genetic background clearly affect the expression patterns. Accordingly, by focusing on genes whose expression pattern is similar between varieties, we can avoid genes that show cultivar-specific light responses.

### Identification of Rice Insertional Mutants Corresponding to Microarray-Derived Candidate Genes

**Figure 1:**
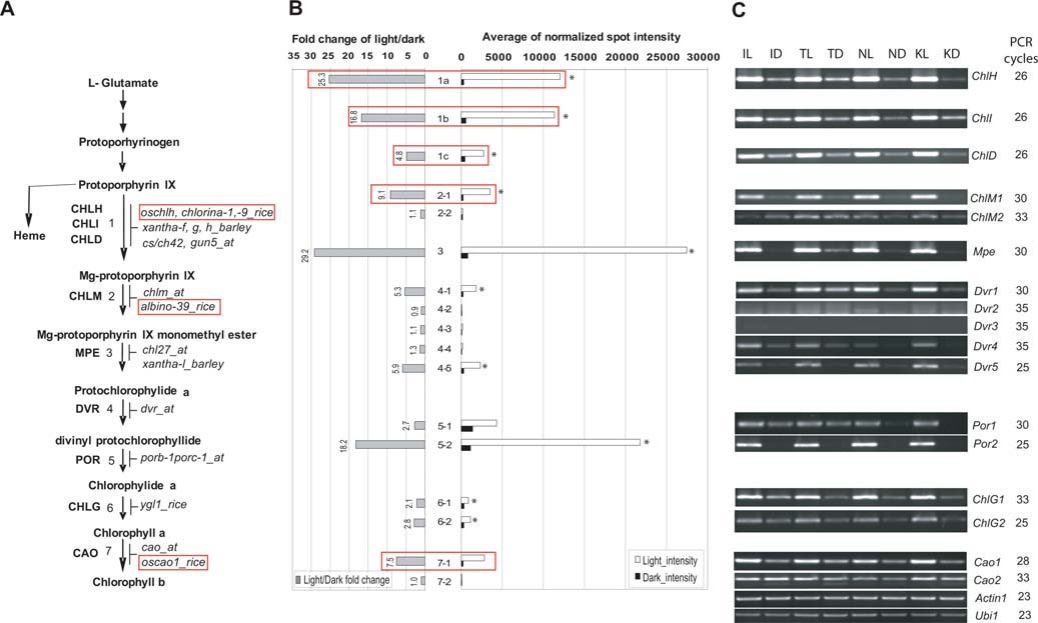
Light-Responsiveness of Rice Genes Involved in the Chlorophyll Biosynthesis Pathway. (A) The seven steps in the rice chlorophyll biosynthesis pathway, from magnesium chelatase to chlorophyll b, are shown and mutants of rice corresponding to each step are indicated. Red boxes indicate mutants which have been characterized in rice. CHLH, magnesium-chelatase subunit H family protein (1); CHLI, magnesium-chelatase subunit I family protein (1); CHLD, magnesium chelatase ATPase subunit D protein (1); CHLM, magnesium-protoporphyrin O-methyltransferase (2, and there were two gene family members); MPE, magnesium-protoporphyrin IX monomethyl ester cyclase (3); DVR, divinyl protochlorophyllide reductase (4, and there were five gene family members); POR, protochlorophyllide reductase (5, and there were four gene family members); CHLG, chlorophyll synthase (6, and there were two gene family members); and CAO, chlorophyll a oxygenase (7, and there were two gene family members). *oschlh*, *chlorina-1*, and *-9* are mutants in rice *ChlH*, *ChlI*, *ChlD*, respectively; *xantha-f*, *g*, and *h* are mutants in barley *ChlH*, *ChlI*, *ChlD*, respectively; *cs/ch42* is mutant in Arabidopsis *CHLI*; *gun5* is mutant in Arabidopsis *CHLH*; *chlm* is mutant in Arabidopsis *CHLM*; *albino-39* is mutant in rice *ChlM*; *Chl27* is mutant in Arabidopsis *MPE*; *xantha-i* is mutant in Barley *Mpe*; *dvr* is mutant in Arabidopsis *DVR*; *porb-1porc-1* is double mutant of Arabidopsis *PORB* and *PORC*; *ygl1* is mutant of rice *ChlG*; *cao* is mutant of Arabidopsis *CAO*; and *oscao1* is mutant of rice *Cao1*. (B) Normalized average expression levels in light and dark and fold changes in expression from dark to light based on results of the NSF45K light *vs.* dark microarray experiment are shown for genes involved in the 7 steps in the pathway and their gene family members. Unique genes or gene family members predominantly expressed in the light in each step are marked with asterisks. White boxes indicate gene expression levels in light. Black boxes indicate gene expression levels in dark. Gray boxes indicate the fold change ratios of light over dark. Red boxes indicate the gene expression patterns of the genes corresponding to those mutations in red boxes of A. *1a*, *Os03g20700*; *1b*, *Os03g36540*; *1c*, *Os03g59640*; *2-1*, *Os06g04150*; *2-2*, *Os02g35060*; *3*, *Os01g17170*; *4-1*, *Os03g22780*; *4-2*, *Os02g56690*; *4-3*, *Os08g17500*; *4-4*, *Os08g34280*; *4-5*, *Os09g25150*; *5-1*, *Os04g58200*; *5-2*, *Os10g35370*; *6-1*, *Os05g28200*; *6-2*, *Os03g09060*; *7-1*, *Os10g41780*; and *7-2*, *Os10g41760*. (C) RT-PCR results are shown; *Ubq1* and *Actin1* were used as controls [85]. Numbers of PCR cycles are indicated. IL, leaves from IR24 plants harvested in the light; ID, leaves from IR24 plants harvested in the dark; TL, leaves of Taipei (TP) 309 plants harvested in the light; TD, leaves of TP309 plants harvested in the dark; NL, leaves of Nipponbare plants harvested in the light; ND, leaves of Nipponbare plants harvested in the dark; KL, leaves of Kitaake plants harvested in the light; and KD, leaves of Kitaake plants harvested in the dark. *ChlH*, *Os03g20700*; *ChlI*, *Os03g36540*; *ChlD*, *Os03g59640*; *ChlM*, *Os06g04150*, *Os02g35060*; *Mpe*, *Os01g17170*; *Dvr1*, *Os03g22780*; *Dvr2*, *Os02g56690*; *Dvr3*, *Os08g17500*; *Dvr4*, *Os08g34280*; *Dvr5*, *Os09g25150*; *Por1*, *Os04g58200*; *Por2*, *Os10g35370*; *ChlG1*, *Os05g28200*; *ChlG2*, *Os03g09060*; *Cao1*, *Os10g41780*; and *Cao2*, *Os10g41760*.

To assess the usefulness of our microarray data we surveyed the expression patterns we obtained through microarray analysis for seventeen genes, including gene family members, which encode proteins for the seven steps in the well-characterized chlorophyll biosynthetic pathway (Figure 1). The expression patterns of all of the candidate genes in the seven steps of this pathway were validated using reverse transcriptase- (RT-) PCR (Figure 1). Based on this analysis we identified four unique genes (Figure 1B, 1a, 1b, 1c, and 3), and two predominantly expressed light-responsive candidate genes (Figure 1B, 2-1 and 7-1) as good targets for studying the biological functions of genes involved in rice chlorophyll biosynthesis. A predominantly expressed light-responsive candidate gene is the one that is most significantly induced by light (compared to other members of the multi-gene family).

Previous studies in rice showed that the knockout mutants of three of the unique genes (encoding the subunits comprising magnesium chelatase complexes in rice: magnesium chelatase subunit H (CHLH, 1a), magnesium-chelatase subunit I family protein (CHLI, 1b), and magnesium chelatase ATPase subunit D protein (CHLD, 1c) exhibited chlorina phenotypes [26],[27]) (Figure S1). The remaining unique gene (magnesium-protoporphyrin IX monomethyl ester cyclase, MPE) at step 3 is predicted to have a function similar to that of its Arabidopsis ortholog, *CHL27* [28].

Knockout lines of the two predominantly expressed light-responsive candidate genes (rice *magnesium-protoporphyrin O-methyltransferase* (*ChlM;Os06g04150*; and gene 2-1) and rice chlorophyll a oxygenase (*Cao1*; *Os10g41780*;and gene 7-1)) had been previously identified and shown to display with light response-related phenotypes [29],[30] (Figure S1). These results indicate that identification of unique genes or the predominantly expressed member of a gene family in the light is an effective method to target genes for further functional analysis. More descriptions on this strategy are shown in Figure S2 and Text S1.

(RiceGE, http://signal.salk.edu/RiceGE/RiceGE_Data_Source.html) [4] (Table 1).

**Table 1:** Rice Insertional Mutant Pools Available and Estimation of Coverage for 365 Light-responsive Candidate Genes Showing at least 8-Fold Induction.

Rice Insertional Mutant Pool	N. of FSTs Mapped a	N. of Genic Regions b	N. of Promoter Sequences c	N. of Mutants d	N. of KO Genes e (%^f^)
PFG T-DNA g	82,520	20,319	7,382	277	161 (45.2)
RTIM Tos17 g	17,937	3,772	695	80	35 (9.8)
RMD T-DNA g	15,610	4,724	1,418	46	34 (9.6)
TRIM T-DNA g	6,965	2,595	743	10	10 (2.8)
Geno-plate T-DNA g	7,187	2,019	582	16	13 (3.7)
ZJ T-DNA g	714	179	42	10	1 (0.3)
CISRO Ac/Ds g	589	311	52	4	4 (1.1)
UCD dSpm/Ds g	10,373	2,419	585	24	21 (5.9)
OSTID Ds g	1,301	751	147	6	6 (1.7)
SHIP Ds g	6,244	1,379	441	20	12 (3.4)
GSNU Ds g	1,046	523	93	3	3 (0.8)
TOTAL g	150,486	26,562	10,585	495	205 (57.6)

a The number of flanking sequence tags (FSTs) mapped based on a total of 57142 gene models released January 24, 2007 at http://www.tigr.org/tdb/e2k1/osa1/pseudomolecules/info.shtml.

b The number of non-redundant genic regions, including exons, introns, and 5′UTRs, in the mutant pool with insertions.

c The number of non-redundant promoter regions in the mutant pool with insertions.

d The number of insertional mutants in each rice insertional mutant pool with insertions in any of the 365 light-responsive candidate genes.

e The number of knocked out (KO) genes corresponding to genic or 5′UTR (within 300 bp upstream from ATG) regions of any of the 365 light-responsive candidate genes in each rice insertional mutant pool.

f The percentage of available knocked out (KO) genes in each rice insertional mutant pool relative to the 365 candidate genes.

g Detailed information on the rice insertional mutant pools was obtained from the Rice Functional Genomic Express Database (http://signal.salk.edu/cgi-bin/RiceGE?DATA=At5g53850).

### Refinement of the Candidate Genes List

**Figure 2:**
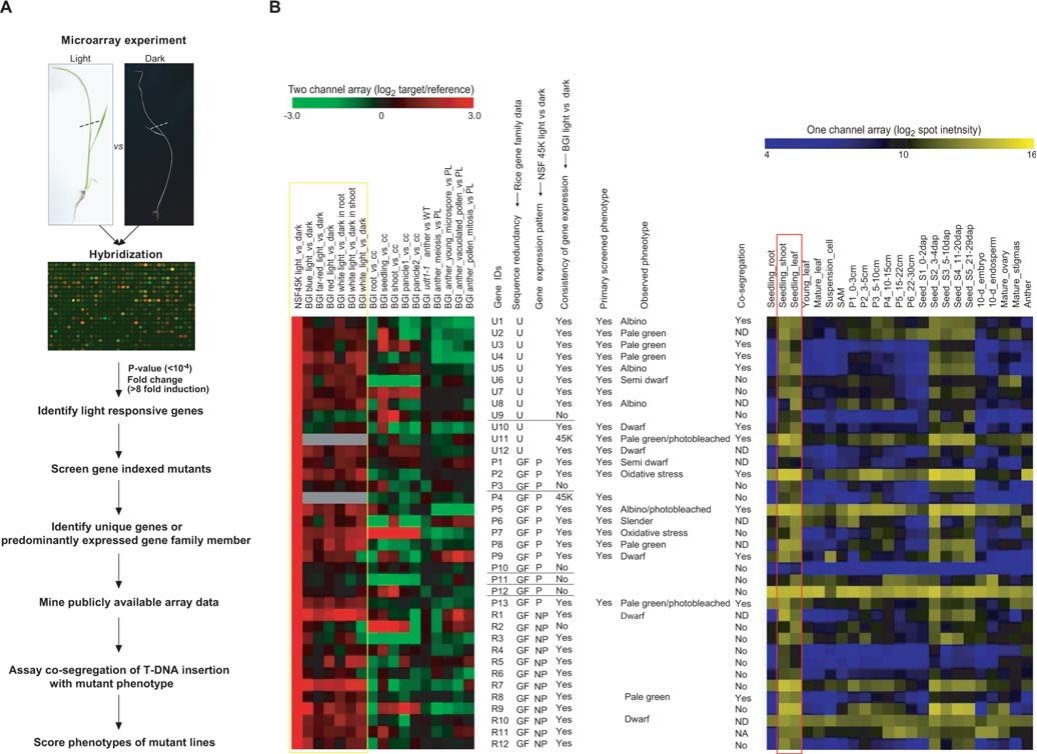
Strategy Used to Refine the List of 37 Candidate Genes Prior to Functional Validation and Summary of Phenotype Analysis. (A) The flow chart of functional analysis using microarray and gene-indexed mutants. (B) Gene expression patterns of selected 37 candidate genes and summary of functional analyses using gene-indexed mutants of these genes. Figure 2A is adapted from a previous review article [4]. Dotted lines in Figure 2A indicate the cut region of rice seedlings used for this array experiment. The NSF45K and BGI/Yale light *vs.* dark array datasets were used to refine the list of 37 candidate genes prior to functional validation. Unique genes (U), genes without gene family members, are distinguished from genes (GF) with gene family members. The gene among the gene family members predominantly expressed in the light was marked as P, distinguished from non predominant genes (NP). The consistency of a gene's expression pattern among the NSF45K and BGI/Yale light *vs.* dark array data was noted with a Yes or No or 45K; 45K indicating the unavailability of any other reference array data for comparison. The genes for which functional analyses was performed are marked Yes. Co-segregation of the observed phenotype and a T-DNA insertion is indicated by Yes, No or ND; ND indicates that co-segregation was not determined due to somaclonal variation or problems with primers. Yellow box indicates rice light *vs.* dark microarray data derived using NSF45K and BGI/Yale array. Red box indicates gene expression data in leaf, seedling shoot, and young leaf derived using the Affymetrix array.

From our NSF45K light *vs.* dark experiment, we selected 365 candidate genes showing at least an 8-fold induction at the 10^−4^ FDR p-value. We then utilized collections of rice insertional mutants to identify lines carrying mutations in the light-responsive genes identified through expression analysis. To date, some 172,500 sequences have been generated from regions flanking insertional mutants in rice and they are publicly available at the Rice Functional Genomic Express Database (http://signal.salk.edu/cgi-bin/RiceGE). Because we wanted to include 2 independently derived mutant alleles in our analysis of each candidate gene so as to help discriminate between phenotypic changes generated by somaclonal variation versus those resulting from the insertional mutations themselves, we limited our phenotypic analysis to 74 mutant lines with T-DNA insertions in a total of 37 candidate genes. The overall scheme we used for functional analysis based on our microarray experiment is presented in Figure 2A and Figure S2).

We classified the 37 candidate genes for which we had corresponding mutants into two groups according to whether the candidate gene belonged to a gene family or not. There were 12 unique genes (those without gene family members) (Figure 2B and Figure S3A) and 25 belonging to gene families (see Materials and Methods). The latter class was further divided into two subgroups by considering the predominance of each gene's expression in the light based on the NSF45K light *vs.* dark array dataset. As a result there were 13 predominantly expressed-light-induced gene family members (referred to as “P” in Figure 2B and as “Predominant”, marked with asterisks in Figure S3B) and 12 gene family members that were not the predominantly expressed in the light (referred to as “NP” marked in Figure 2B and as “Non predominant”, marked with sharps in Figure S3C).

Because other more predominantly or equally expressed gene family members might compensate for a defective gene family member in the light, the non-predominantly expressed gene family members were not considered good candidates for functional analysis and were initially excluded from the functional analysis (Figure S3C). Next, we identified 5 genes, *Os03g48030*, *Os11g05050*, *Os02g58790*, *Os09g37620*, and *Os09g16950*, for which light responses between the NSF45K and BGI/Yale light *vs.* dark array datasets were significantly inconsistent and deleted them from our primary list of candidate genes for the initial round of functional analysis (Figure 2). Of these, *Os03g48030* was a unique gene and *Os11g05050*, *Os02g58790*, *Os09g37620*, and *Os09g16950* were the members of their respective gene families the most predominantly expressed in the light in the NSF45K array data set.

**Figure 3:**
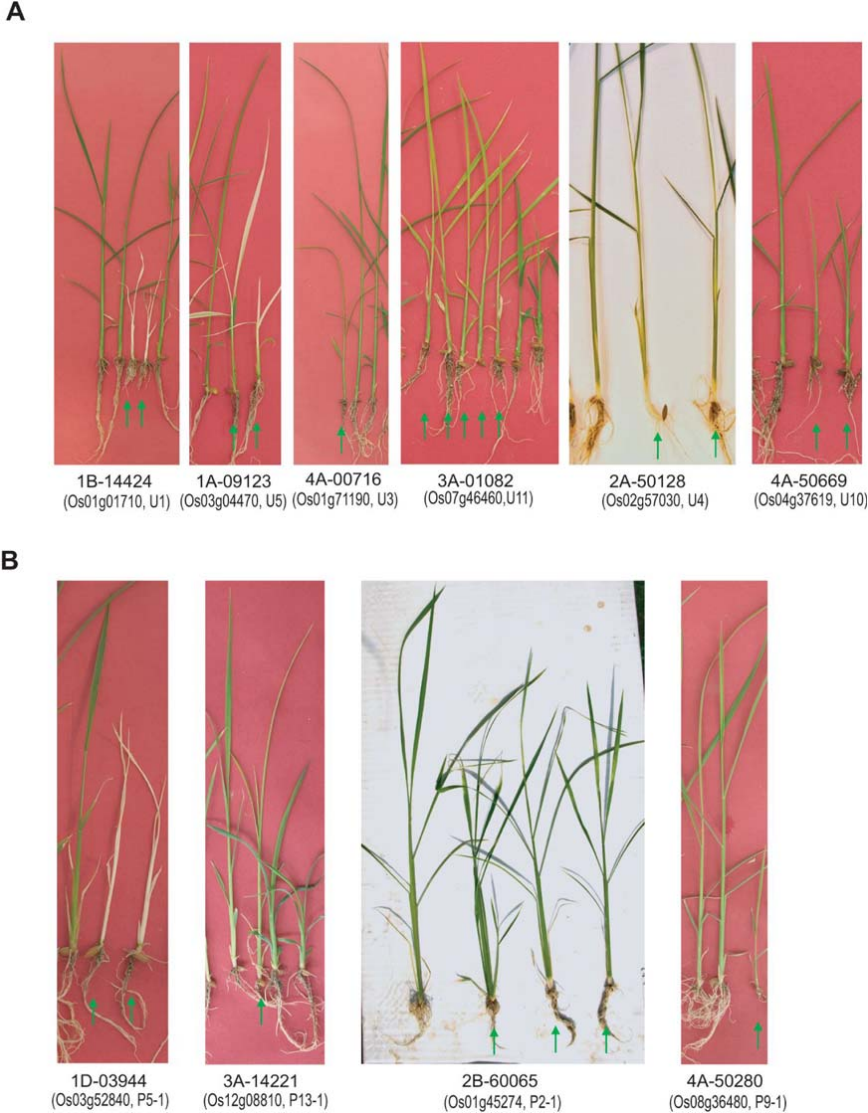
Functional Analyses of Unique Genes and Gene Family Members Predominantly Expressed in the Light by Using T-DNA Insertional Mutants. (A) Co-segregating phenotypes observed in knockout lines affecting 6 unique genes. (B) Co-segregating phenotypes observed in knockout lines affecting 4 gene family members predominantly expressed in the light. Green arrows indicate mutant phenotypes that co-segregated with T-DNA insertions in the candidate gene (gene designations are given in parentheses). All primers used for genotyping are described in Table S5. The phenotype analyses for 20 light-inducible genes are detailed in Table 8.

We also included one unique gene (*Os07g46460*) and one predominantly light-induced gene family member (*Os03g37830*) in our candidate gene list based only on our own data because information on their light responses was not available among the BGI/Yale data (Figure 2). We then screened the remaining mutants, those associated with 11 unique genes and 9 predominantly light-induced gene family members, to determine their phenotypes (Figure 3). We also assayed the phenotypes of the knockout lines associated with the 17 genes we had eliminated from our primary list of candidate genes to check the efficiency of identifying mutant phenotypes for the not predominantly light-induced genes in a gene family and/or genes with expression patterns that weren't consistent between the NSF45K and BGI/Yale light *vs.* dark array datasets. (Detailed data regarding all 37 of the genes that were functionally analyzed are presented in Table S2.) Our criteria for selecting candidate genes that respond to light might have inadvertently eliminated from consideration genes among the BGI/Yale data that exhibit condition-dependent light responsiveness.

### Functional Analysis of 20 Candidate Genes

We initially carried out functional analysis for 20 selected candidate genes, 11 unique genes and 9 predominantly light-induced gene family members (Figure 3A and 3B). First, we identified defective phenotypes associated with six of the 11 unique genes that we analyzed for function from our list of top candidate candidates. Of these, the phenotypes of knockout lines associated with *Os01g01710* (*1-deoxy-D-xylulose 5-phosphate reductoisomerase*, *Dxr*) and *Os03g04470* (*Expressed protein*) were albino and displayed chlorotic leaves, respectively, 2 weeks post-sowing (Figure 3A). Knockouts of *Os01g71190* (*Photosystem II subunit 28*, *Psb28*), *Os02g57030* (*Expressed protein*), and *Os07g46460* (*ferredoxin-dependent glutamine:2-oxoglutarate aminotransferase*, *Fd-GOGAT*) displayed pale green phenotypes (Figure 3A). Of these, a mutation in the *Fd-GOGAT* gene displayed photo-bleached leaves two weeks later after revealing pale green leaves (Data not shown). Knockouts of *Os04g37619* (*Zeaxanthin epoxiydase*, *Aba1*) produced dwarf mutants (Figure 3A).

We noted that the mutant phenotypes associated with three of these 6 genes, those encoding DXR, Fd-GOGAT, and ABA1, were similar to those associated with their Arabidopsis orthologs [31],[32],[33]. The Arabidopsis ortholog of *Os01g71190* encodes photosystem II (PSII) reaction center Psb28 protein (Psb28) that was first identified from PSII of Synechocystis 6803 with Psb27 [34]. The function of this PSB28 has not yet been well- characterized, although it is predicted to serve a role as a regulatory protein based on its substoichiometric amount [34],[35]. Similarly, Arabidopsis lines carrying a mutation in *Psb27* did not display a severe phenotype. Recovery of PSII activity after photoinhibition was delayed in the Arabidopsis *psb27* mutant supporting a role in PSII for this gene [36]. The mild phenotype displayed by the *Psb28* T-DNA insertional rice plants in *Psb28* gene suggests that this gene product might serve as a regulatory protein to stabilize PSII activity. The other two unique genes for which we identified corresponding mutant phenotypes as a result of our analysis encode as yet unidentified proteins.

The endogenous retrotransposon *Tos17* has been shown to be an efficient insertional mutagen in rice and phenotypes of some 50,000 M2 generation insertion lines carrying *Tos17* insertions have been reported [21]. We used the available *Tos17* phenotypic data to assess the functions of some of our candidate genes. Phenotypes similar to those identified in the T-DNA insertional mutants for *Os01g71190* (*Psb28*), *Os03g47610* (*Dxr*), and *Os04g37619* (*Aba1*) have also been observed for the corresponding *Tos17* insertional mutant lines (Table 2; [21],[37]).

**Table 2:** Summary of Screen for Phenotypes Associated with 20 Candidate Genes Showing at least 8-Fold Induction in the Light.

T-DNA					*Tos17*
Locus_ID^a^ (Gene ID b)	Annotation	Line^c^	Phenotype^e^	Co^d^	Line^c^/phenotype
**Unique genes screened**					
Os01g01710 (U1)	1-deoxy-D-xylulose 5-phosphate reductoisomerase	1B-14224	albino	O	
		1C-03301	albino	O	
Os01g40710 (U2)	Expressed protein	3A-00098	NA	LS	
		3A-08586	pale green	ND	
Os01g71190 (U3)	Psb28	4A-00716	pale green	O	ND6028/ dwarf
		1C-06727	NA	LS	
Os02g57030 (U4)	Expressed protein	2A-50128	pale green	O	
		4A-50119	pale green	O	
Os03g04470 (U5)	Expressed protein	1A-09123	albino	O	
		3A-03564	albino	ND	
Os03g06230 (U6)	Expressed protein	1B-01520		X	
		4A-00253		X	
Os03g19760 (U7)	HAD-superfamily hydrolase	1B-01342		X	
		1C-06809		X	
Os03g47610 (U8)	ThiC	1A-08723	NA	No homo	NF6834/ albino
		1A-17021	NA	No homo	
Os04g37619 (U10)	Zeaxanthin epoxidase	3A-16669	dwarf	O	ND7057/ dwarf
		4A-50669	dwarf	O	
Os07g46460 (U11)	Ferredoxin-dependent glutamate synthase	1D-02443	pale green	O	
		3A-01082	pale green	O	
Os08g40160 (U12)	Thylakoid lumen protein	2B-20208	dwarf	ND	
		2B-20162	dwarf	ND	
**Predominant gene family members screened**					
Os01g08460 (P1-1)	TMS membrane family protein	2B-00356	Semi-dwarf	ND	ND7938/semi_dwarf
		1B-20710	Semi-dwarf	ND	ND1302/ semi_dwarf
Os01g45274 (P2-1)	Carbonic anhydrase	2B-60065	oxidative stress	O	
		3C-00294	oxidative stress	O	
Os03g37830 (P4-1)	Potassium uptake protein	2C-10006	albino	X	
		2D-40669	albino	X	
Os03g52840 (P5-1)	Serine hydroxylmethyl transferase	1D-03944	albino	O	NC2658/ albino
		3A-60218	NA	LS	NC3521/ albino
Os05g47540 (P6-1)	Phosphoethanolamine N-methyltransferase	3A-07919	slender	ND	NC6571 /albino
		4A-00172	NA	LS	ND4332 / albino
Os06g04510 (P7-1)	Enolase 1	3A-02083	pale green	X	NF2004 / hard leaf
		3A-12320	pale green	X	
Os07g32880 (P8-1)	ATP synthase gamma chain	2A-10285	NA	No homo	
		3D-01688	pale green	ND	
Os08g36480 (P9-1)	Nitrate reductase 1	2D-10946	NA	No Homo	
		4A-50280	dwarf	O	
Os12g08810 (P13-1)	VTC2	3A-14221	pale green	O	
		3A-50953	NA	No homo	

a TIGR Locus identifiers.

b Gene identifiers used in Figure 2, Figure 3, Figure 4, Figure 5, Figure S3, Figure S4, Figure S5, and Figure S7.

c Name of line with T-DNA or *Tos17* insertion in the candidate gene [21]. (In most cases, two independent lines, and therefore two independent alleles, were selected for characterization.)

d Results of tests for co-segregation of insertion and phenotype. O, a co-segregating line; X, a non co-segregating line; ND, co-segregation not determined due to issues involving somaclonal variation or problems with designed primers; No homo, there were no homozygous progenies; and LS, less seeds due to the very low fertility.

e NA, not analyzed because homozygous progenies were not available for screening or seeds were not available due to the very low fertility.

We could not identify mutant phenotypes for the other unique genes containing T-DNA insertional mutations that we analyzed. We assume that phenotypes associated with two genes, *Os01g40710* and *Os08g40160*, could not be determined due to confounding somaclonal variations (Figure S4) in the one mutant line available for each of these genes for analysis. We could not analyze the second mutant line corresponding to each of these genes due to poor seed set. Lines with T-DNA insertions in *Os03g47610*, lines 1A-08723 and 1A-17021, did not produce any homozygous progenies and as a result the phenotypes associated with these mutations were also not determined (Table 2; Table S2). On the other hand, while we did identify homozygous progenies and their siblings among mutants with insertions in *Os03g06230* and *Os03g17960*, we did not observe phenotypic differences between them (Table 2).

Targeting functional analysis to unique genes is an effective way to significantly increase the efficiency of identifying genes corresponding to defective phenotypes. Utilizing microarray-derived expression profiles of unique genes can increase the efficiency of functional analyses [1],[22],[38]. The efficiency (6/11) with which we were able to identify phenotypes associated with mutations in unique genes demonstrates the power of combining knowledge of gene copy number and gene expression patterns.

Of the nine predominantly light-induced gene family members that were consistently induced in the light, we found four for which mutant phenotypes co-segregated with a T-DNA insertion in the gene (Figure 3B). These four genes encode carbonic anhydrase 1 (CA1), serine hydroxymethyltransferase 1 (SHMT1), nitrate reductase 1 (NR1), and Vitamin C defective 2 (VTC2), respectively. The rice T-DNA insertional line carrying a mutation in the *Shmt1* gene (*Os03g52840*), line 1D-03944, displayed variegated chlorina leaves (Figure 3B). The line 2B-60065 with a T-DNA insertion in *Ca1* (*Os01g45274*) showed an oxidative stress-related phenotype, necrosis in the middle of the leaf, and a little growth retardation (Figure 3B). Line 4A-50280, with a T-DNA insertion in *Nr1* (*Os08g36480*), displayed dwarfism, and line 3A-14221, with a T-DNA insertion in the *Vtc2* gene (*Os12g08810*), exhibited a pale green and later photo-bleached leaves phenotype (Figure 2B). The *Tos17* line with an insertion in the rice *Shmt1* gene exhibited the same phenotype as the corresponding T-DNA mutant, line 1D-03944 (Table 2). The phenotypes associated with mutations in *Shmt1* (*Os03g52840*), *Nr1* (*Os08g36480*), and *Vtc2* (*Os12g08810*) were also reminiscent of the phenotypes associated with mutations in the orthologous genes in Arabidopsis [39],[40],[41],[42]. Mutant phenotypes associated with T-DNA insertions in *Os01g08460*, and *Os05g47540* were not determined due to confounding somaclonal variations (Figure S4). The homozygous progenies of mutant lines with insertions in two other genes, *Os03g37830* and *Os06g04510*, did not show visible phenotypic changes (Table 2).

Our success in conducting functional analyses of gene family members that are the predominantly expressed in the light, and consistently so from one microarray experiment to the next, suggests that the functions of gene family members can be successfully analyzed by utilizing data obtained using microarrays representing nearly complete plant transcriptomes. Analyses that consider both sequence similarity and predominance of gene expression has also been reported to be quite effective in functional analysis of yeast genes [14].

### Functional Analysis of 17 Non-prioritized Candidate Genes

We also carried out functional analysis of 5 genes that showed inconsistent gene expression patterns when we compared our NSF45K light-response experiments and the BGI/Yale light *vs.* dark experiments. One of them, *Os03g48030* (designated U9 in Figure 2 and Figure 3), is a unique gene and the other four genes, encoding lectin protein kinase (*Os09g16950*), stem-specific protein TSJT1 (*Os11g05050*), flavin-containing monooxygenase family protein (*Os09g37620*), and an expressed protein (*Os02g58790*), were the predominantly light-induced members of their respective families among the NSF45K array-derived data but not in the BGI/Yale light *vs.* dark dataset. No defective phenotypes were observed among the mutant lines with T-DNA insertions in any of these genes (Table S3). One possible reason for this result is the presence of redundant metabolic networks or the absence of appropriate screening conditions [15],[43],[44].

Mutants carrying insertions in 12 genes that were not the predominantly expressed light-induced member of their respective gene family were also examined in this study. A visible phenotype was observed in the homozygous mutant progenies associated with only one of these genes, *Os07g05000* (*R8-2*) (Figure S3C and Figure S5), which belongs to the family of genes encoding aldo/keto reductases. Phenotypic changes were not observed in the homozygous segregants of lines with mutations in any of the other genes (Table S3). The absence of detectable abnormal phenotypes associated with these other genes is generally believed to be due to one (or more) family members compensating for the function of the mutated gene [14],[15]. Line 3A-03008, which carries a T-DNA insertion in *Os07g05000*, showed a weakly pale green phenotype and slight growth retardation (Figure S5).

Identification of phenotypes associated with the other mutations may require specific conditions under which there will be no compensatory gene expression from other family members. In cases of gene families without a predominantly expressed member under specific experimental conditions, microarray data can still be used to identify the multiple significantly expressed genes in a family so that they can be subjected to RNA-silencing techniques as has been carried out by Miki et al. [18],[45] for the rice genes encoding homologs of mammalian Rac GTPase, *OsRac1* and *OsRac5*. Of our list of non-predominantly light-induced genes, there were two genes from same family expressed under light conditions (*Os12g03070* and *Os11g03390*). Both encode an FHA domain, which is a putative nuclear signaling domain found in protein kinases and transcription factors. However, we did not observe a phenotype in knockout lines of *Os11g03390* (*R12-1*) [46] (Table S3). Similarly, we did not observe phenotypic changes associated with T-DNA insertions in members of the gene families encoding ABC1 proteins (*Os02g57160*, *R3-1* and *Os04g54790*, *R5-1*), S1-RNA binding domain proteins (*Os04g54790*, *R4-1*), and glycine dehydrogenases (*Os06g40940*, *R7-1*). Further experiments to generate double mutants for these family members and their light-induced relatives will be required to elucidate their functions. In Arabidopsis, the light-responsive functions of genes in gene families such as *POR* and *PHOT* have been clarified by using double mutants of two family members, *porbporc* and *phot1phot2*, respectively [47],[48].

When we consider severity of phenotypes, two of six lines carrying defects in unique genes and one of four lines carrying defects in the gene family member predominantly expressed in the light died at early seedling stage (Figure 3). Therefore, these three genes are essential for survival.

Overall, phenotypes of mutants with defects in unique genes were more frequently observed than those of mutants carrying defects in predominantly light-induced gene family members (compare Figure 3A with Figure 3B). We suspect, therefore, that other members in a gene family may carry out a somewhat compensating role [14],[15]. Similarly, we did not find phenotypic changes associated with mutations in genes that were not the predominantly light-induced members of their respective families, with the exception of mutations in a gene encoding an aldo/keto reductase protein. This result also supports our hypothesis that other gene family members can compensate for the mutation. Despites these observations, we can not rule out the possibility that the absence of phenotype is due to non-optimal environmental conditions [44].

In summary, we identified phenotypic changes in rice lines carrying mutations in 10 out of 20 unique genes or genes that were the most predominantly light-induced members of their respective families. In contrast, we discovered only one phenotypic change among the lines carrying mutations in the 17 other genes that either showed inconsistently light-induced expression among different microarray data sets or were not the predominantly light-induced members of their respective gene families (Table S3). Microarray data were very useful as criteria for prioritizing candidate genes for functional analyses. Consideration of the expression patterns of all the genes within a gene family is an effective way to approach study of the functions of gene family members [14].

### Validation of Our Strategy using Arabidopsis Functional Profiling Data

Functional profiling of genes related to the phytochrome-mediated signaling pathway in Arabidopsis was recently carried out [3]. We used this data set to further test the usefulness of our method (see Materials and Methods). Thirty two genes were selected for this functional profiling analysis. Of these, mutants in seven genes displayed statistically significant photomorphogenic phenotypes. Except for one gene (*At2g46970*) whose gene expression profiling data was not available, we found that six genes were either unique sequences or the predominantly expressed gene family member in the red light. In contrast, mutations in the remaining 25 genes showed less significant photomorphogenic phenotypes; thirteen of them displayed mild or severe defects in photoresponsiveness and 12 did not showed distinguishable phenotypes (Figure S6) [3]. Of these, 10 genes were not the predominantly expressed gene family member in the red light whereas 13 genes (except two genes; *At3g21550* and *At3g21330* whose gene expression profiling data was not available) were either unique sequences or the predominantly expressed gene family member in the light (Figure S6). Thus, this functional profiling analysis in Arabidopsis also indicates that predominantly expressed gene family members as well as unique genes are good targets for functional validation.

### Identification of Relationships among 13 Biochemical Pathways

**Figure 4:**
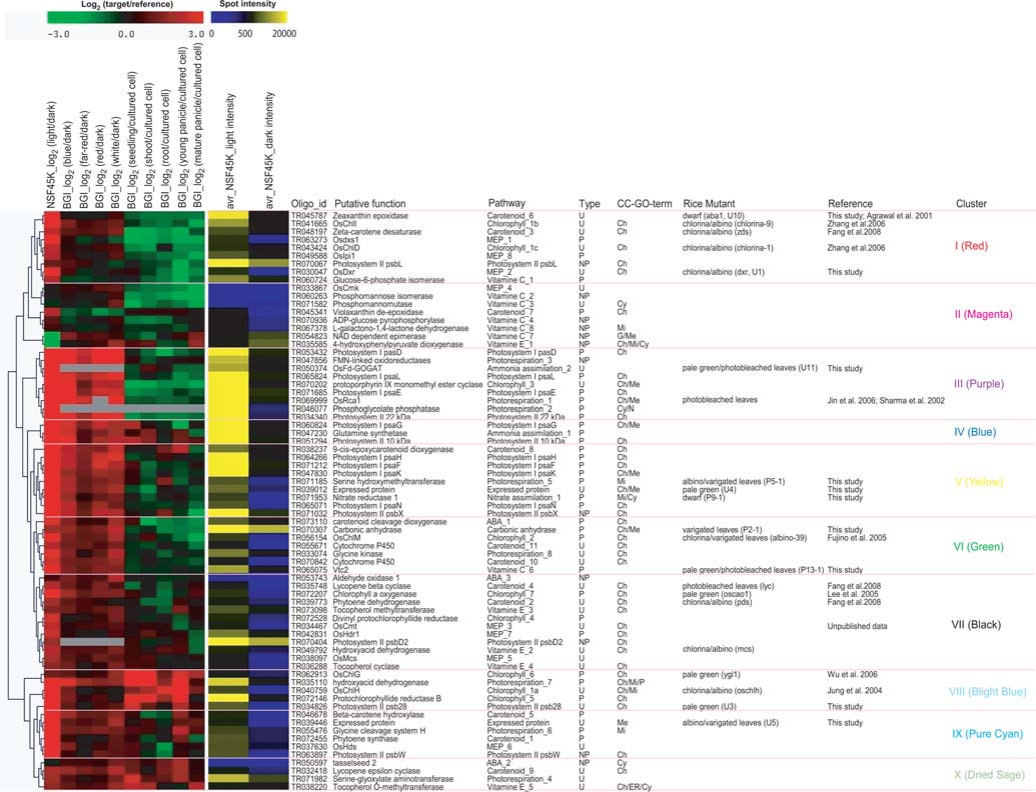
Co-Expression Analysis of 72 Genes in the Pathways Related to 10 Mutants Identified in This Study. Hierarchical clustering analysis was carried out for 72 genes related to the 10 mutants shown in Figure 3; cluster results are indicated on the far left side of this figure. In the left panel, the log_2_ fold change values of the 10 datasets used to perform hierarchical clustering analysis are shown (for details of the 10 datasets and how they were analyzed see Materials and Methods). The middle panel indicates the average of all spot intensities for an oligo in the light (av_NSF45K_light intensity) and dark (av_NSF45K_dark intensity) from NSF 45K light *vs.* dark datasets as another indicator of gene expression levels. Oligo_id indicates the name of the oligos in NSF45K array; Putative function describes the annotation assigned to that gene; Pathway indicates the biochemical pathway and step in the pathway associated with the gene; Type indicates status of the gene in the rice genome: U, unique sequence; P, predominantly expressed gene family member in the light; and NP, not the predominantly expressed gene family member in the light; CC-GO_term, indicates GO terms within the cellular component category; Rice Mutant indicates the phenotype associated with a mutation in the gene; and Reference provides citations to published evidence for the phenotypes described in the previous column. More information regarding U1, U3, U4, U5, U10, U11, P2-1, P5-1, P9-1, and P13-1 is contained in Table 2. In the column labeled CC-GO_term: Ch indicates chloroplast; Cy, cytoplasm; ER, endoplasmic reticulum; Mi, mitochondrion; Me, membrane; N, nucleus; and P, peroxisome. Data used for clustering analysis and more detailed information regarding the 72 genes on which the analysis was performed are contained in Table S4.

Mutant phenotypes clearly suggest functions for targeted genes and also for the pathways those genes are associated with. Therefore, understanding relationships among multiple pathways containing mutants defective in the plant's response to light will help us elucidate the light response. To do this, we identified genes among various pathways that were co-expressed with the 10 genes for which we had identified mutant phenotypes in this study. We found that ten pathways (http://www.gramene.org/pathway/) involved 7 of the genes for which we had identified mutants (Figure 4; Table S4). Sixty nine genes in these 10 pathways were selected as described in Materials and Methods. Additionally, three single-step reactions unlinked to any of these pathways but involving the other three genes for which we identified mutant phenotypes in this study, U4 (*Os02g57030*), U5 (*Os03g04470*), and *Ca1* (P2-1, *Os1g45274*) were also included in this analysis (see Table 2).

All together, 72 genes involved in the 10 pathways and three reactions were selected for hierarchical clustering analysis (Table S4). Then, we selected 10 datasets with which to carry out the analysis (Table S4): log_2_ fold change values of NSF45K light *vs.* dark and four different types of light *vs.* dark datasets generated by BGI/Yale array [49], and log_2_ fold change values of five different tissues *vs.* cultured cells [50]. We selected candidate genes in each pathway that are unique or are the predominantly light-induced gene family member (except several steps not represented by predominantly light-induced gene family members). We found that gene expression patterns of 67 out of the 72 genes are light-inducible in at least two of the light treatments (Figure 4). Fifty-five genes have GO terms in the cellular component category and 46 of them have a chloroplast GO term in the cellular component category. Seven genes are predicted to have role in the mitochondrion (Figure 4). Most of the genes used for the clustering analysis are predicted to perform their light response-related function in chloroplasts or mitochondria (Figure 4). As a result of the hierarchical clustering analysis we identified 10 gene clusters (Figure 4). As has been previously reported [50],[51],[52], co-expression analysis is useful for revealing functionally coherent groups of genes.

We next looked for relationships among different pathways by utilizing Cytoscape software to analyze the results we obtained from our co-expression analysis (see Materials and Methods). Cytoscape is an open source software for integrating biomolecular interaction networks with high-throughput expression data [51]. The results of this analysis are shown in Figure S7.

**Figure 5:**
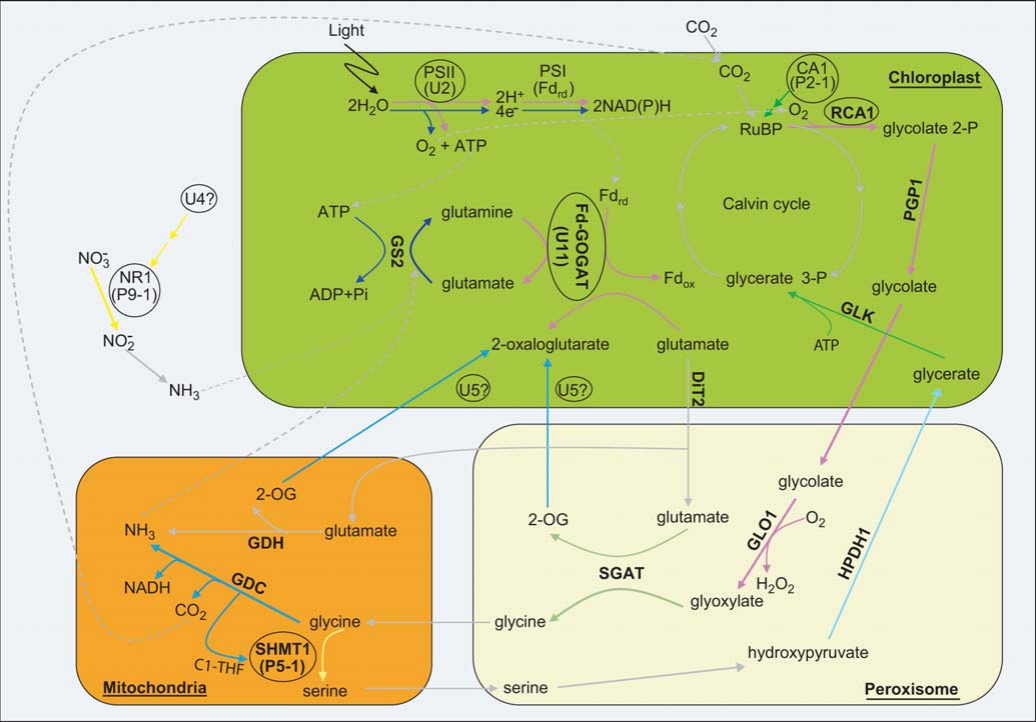
Schematic Representation of the Role of Rca1, Shmt1, U4, U5, Nr1, Ca1, and Psb28 in Photorespiration, Ammonia Assimilation, Nitrate Assimilation, and Photosynthetic Light Reaction Pathways. Photorespiration occurs in three organelles: the chloroplast, the peroxisome, and the mitochondria. Ammonia association pathway is tightly linked to photorespiration for recycling ammonia, byproduct of photorespiration. Rubisco activase 1 (Rca1) commits photorespiration by helping to incorporate O_2_ to active site of ribulose bisphosphate carboxylase/oxygenase (RUBISCO) and ribulose bisphosphate (RuBP) is oxidized to 3-phosphoglycerate (glycerate 3-P) and 2-phosphoglycolate (glycolate 2-P). Then, glycolate 2-P is converted to glycine by a series of reactions: 2-phosphoglycolate phosphatase1 (PGP1), glycolate oxidase (GLO1) serine-glyoxylate aminotransferase (SGAT). The decarboxylation of two glycines produces serine, CO_2_ and NH_3_ by glycine decarboxlasse complex (GDC) and serine hydroxymethyltransferase 1 (SHMT1, P5-1). In addition, gulatamate dehydrogenase (GDH) in mitochondria deaminates glutamate and generates 2-oxoglutarate (2-OG) and NH_3_ needed for ammonia assimilation (GS2/Fd-GOGAT cycle). SGAT also generates 2-OG from glutamate. Glutamate is supplied from chloroplast by glutamine synthase 2 (GS2) and ferredoxin dependant glutamine-oxoglutarate aminotransferase (Fd-GOGAT, U11) in ammonia assimilation, and glutamate/malate transporter (DiT2). Function of U5 gene product is suggested to transport 2-oxoglutarate to chloroplast. Serine is further converted to 3-phosphoglycerate by hydroxypyruvate dehydrogenase (HPDH1) and glycerate kinase (GLK). Photorespiration consumes CO_2_ and energy (ATP) in photosynthetic cells. Photosynthetic light reaction carried out by photosysntem I (PSI) and photsystem II (PSII, U3) supplies O_2_, ATP and reduncing powers (reduced ferredoxin form, Fd_rd_). Nitrate (NO_3_−) is another source of nitrogen for plant growth and nitrate assimilation pathway is alternative way to provide ammonia for GS2/Fd-GOGAT cycle. Nitrate reductase 1 (NR1, P9-1) commits this pathway. U4 is suggested to have roles in transcriptional regulation of nitrate assimilation pathway. Glycerate 3-P resulted from photorespiration goes into Calvin cycle for the CO_2_ fixation. Carbonic anhydrase 1 (CA1, P2-1) might be involved in CO_2_ fixation in RuBP. Black circles indicate the steps in the pathway which are related to mutants identified in this study. Question marks (?) indicate genes of unknown function (*U4* and *U5*). Purple lines indicate genes belonging to co-expressed cluster III; blue, cluster IV; yellow, cluster V; bright blue, cluster VIII; pure cyan, cluster IX; and dried sage, cluster X in Figure 4. Weak gray lines indicate steps in each pathway not associated with data collected in this study.

First, cluster III (purple lines in Figure 4 and Figure S7) contained 3 components of PSI, one of PS II, three from the photorespiratory pathway, one from the ammonia assimilation pathway, and one from the chlorophyll biosynthetic pathway. Of these, ferredoxin-dependent glutamine:2-oxoglutarate aminotransferase (Fd-GOGAT, U11) couples with glutamine synthetase 2 (GS2) for assimilating ammonium produced by photorespiration [33],[52]. In Figure 4 and Figure 5, the *Fd-GOGAT* gene (*U11*), which generates glutamate at step 2 of the ammonia assimilation pathway is co-expressed with the PS I and PS II components Os08g44680, Os12g23200, Os07g25430, and Os01g64960. GS2 (OS04g56400) at step 1 of this pathway is co-expressed with other PS I and PS II components (Os09g30340 and Os08g10020). PS I and PS II supplies ATP for the reaction of GS2 and reduced ferredoxin (Fd_rd_) for Fd-GOGAT [53]. This dependency of the ammonia assimilation pathway on photosynthesis is supported by the co-regulation of several PS I and PS II components with two genes in the ammonia assimilation pathway (Figure 4, Figure 5, and Figure S7).

The photorespiratory pathway is known to be tightly correlated with nitrogen cycles [54]. Co-expression patterns of genes encoding three early steps of the photorespiratory pathway with the *Fd-GOGAT* gene suggest a close relationship of photorespiration with the ammonia assimilation pathway (purple lines in Figure 4, Figure 5 and Figure S7). This makes sense because the ammonia assimilation pathway plays a role in preventing loss of ammonia, which is generated from step 6 of the photorespiratory pathway by refixing it to glutamate [53],[55],[56]. Furthermore, the resulting amino acid (i.e. glutamate) reacts with glycine to synthesize glutathione in the peroxisome, suggesting that this pathway is important for protecting photosynthetic apparatus (e.g. PS II) from toxins such as free radicals [55]. Also, co-regulation of genes in the three early steps of the photorespiratory pathway with several PS I and II components suggests that oxygen generated by photosynthesis triggers the photorespiration pathway [57],[58]. Co-regulation of genes involved in photosynthesis, ammonia assimilation and photorespiration can explain their coordinated functions [56].

Of the genes in cluster III, the phenotypes of a mutant line with a T-DNA insertion in *Fd-GOGAT* (*U11*) and a mutant lines under-expressing the *Rca1* gene (*Os11g47970*) were characterized (Figure 4, Figure 5 and Figure S7) [59]. The *Rca1* under-expressed mutant displays chlorotic leaves [59]. The mutant (3A-01082, this study) with a T-DNA insertion in *Fd-GOGAT* gene displayed pale green leaves shortly after germination (Figure 3). The same mutant displayed chlorotic leaves four weeks after germination and is similar to the phenotype of one of the *Rca1* under-expressed mutants (data not shown) [59].

These results indicate that co-expressed groups of genes carry out closely related functions as reported in other species [1],[33],[60]. Therefore we can predict that mutations in other genes in this cluster will display similar phenotypes to those observed in the *Fd-GOGAT* and *Rca1* mutant lines. Those predicted to display such phenotypes include phosphoglycolate phosphatase (*Pgp*, *Os04g41340*) [61] at step 2 of the photorespiration pathway. In support of this hypothesis, a mutant with defects in Arabidopsis (*PGLP1*) displayed chlorotic leaves at the early seedling stage, the phenotype was similar to that observed for *Rca1* and *U11* mutant lines [61].

#### Assigning Functions of Genes Unlinked to Known Biochemical/Metabolic Pathways Function of U5 (Unknown Gene)

Gene ontology can help assign putative functions to unknown genes [62],[63]. For example, Os03g04470 (unknown gene, U5) has a sodium/dicarboxylate cotransporter activity GO term (GO:0017153) in the molecular function category. Because it is also assigned a membrane GO term (GO: 0016020) in the cellular component category, the U5 gene product is likely to be membrane-bound. The nearly identical expression pattern of the *U5* gene with the *glycine cleavage system H* gene (*Gcsh*, *Os10g37180*) encoding a glycine decarboxylase complex H protein subunit in step 6 of the photorespiration pathway suggests that the *U5* genes may function as a chloroplast sodium/dicarboxylate transporter[64]. In support of this hypothesis, a mutation in the *U5* gene causes a defect in transporting 2 oxaloglutarate, and blocks re-assimilation of ammonia generated by the photorespiratory cycle. This result suggests that the supply of glutamate needed for synthesis glycine by serine-glyoxylate aminotransferase (SGAT, step 4 of photorespiration pathway) is limited. The U5 mutant line displays variegated albino leaves at the early seedling stage and later becomes albino. This phenotype is similar to that observed for a mutation in the gene controlling step 5 (SHMT1, P5-1) of the photorespiration pathway (Figure 3 and Figure 5). A phenotype exhibited by lines carrying mutations in the *U5* and *Shmt1* (*P5-1*) genes suggests that blockage of 2-OG transport by the defect in the *U5* gene causes a shortage of glycine, which is a substrate for serine biosynthesis controlled by the P5-1 gene product (Figure 5) [42].

#### Function of U4 (Unknown Gene)

Based on its GO terms (GO:0009579 and GO:0006355), *Os02g57030* (*unknown gene*, *U4*) is predicted to be located on the thylakoid membrane and carry out transcription. The *nitrate reductase 1* gene (*Nr1*, *Os08g36480*) controlling step 1 of the nitrate assimilation pathway displays an expression pattern most similar to that of *U4* (in Figure 4 and 5). Thus a probable role for U4 is to carry out light-dependent transcription regulation of nitrate assimilation (Figure 4 and Figure 5).

#### Function of CA1 Unlinked to Known Pathways

Unlike the above two genes, which previously had no known function, the *Ca1* gene (*P2-1*, *Os01g45274*) is predicted to catalyze the reversible conversion of bicarbonate (HCO_3_^−^) to CO_2_ [65]. However, CA1 is still not directly linked to the known biochemical/metabolic pathways shown in Figure 5 and Figure S7. We have now determined that *Ca1* (*P2-1*) is co-expressed with *glycerate kinase* gene (*Glk*, Os01g48990) that controls step 8 of the photorespiratory pathway (Figure 5 and Figure S7). This result suggests that rice CA1 carries out roles associated with RubisCO (ribulose-1,5-biphosphate carboxylase/oxygenas) and the Calvin cycle as reported in Cyanobacterium and Algae [65],[66].

### Transcriptional Hierarchy of Genes in the MEP Pathway

The MEP pathway is a unique and essential process for plants, algae and bacteria [67],[68],[69]. The final metabolites of the pathway are isopentenyl pyrophosphate (IPP) and its isomer dimethylallyl pyrophosphate (DMAPP), which are used for the synthesis of isoprenoids (such as isoprene), carotenoids, plastoquinones, phytol conjugates (such as chlorophylls and tocopherols), and hormones (such as gibberellins and abscisic acid) [60],[70],[71],[72],[73],[74].

Co-expressed Cluster I consists of genes (*Os05g33840*, *Os07g36190* and *Os05g33840*) controlling steps 1, 2, and 8 of the MEP pathway, genes (*Os04g37619* and *Os07g10490*) controlling steps step 3 and 6 of the carotenoid biosynthetic pathway, genes (*Os03g36540* and *Os03g59640*) encoding two components (CHLI and CHLD) controlling step 1 of the chlorophyll biosynthetic pathway, gene (*AK059143*) encoding Psba of photosystem II, and gene (*OS09g29070*) controlling step 1 of the vitamin C biosynthetic pathway. Co-expression of genes in the MEP pathways with those in the chlorophyll and carotenoid biosynthesis pathways supports the hypothesis that the syntheses of pigments mediated by metabolite(s) resulted from the MEP pathway are dependent on photosynthesis (Figure 4 and Figure S7).

**Figure 6:**
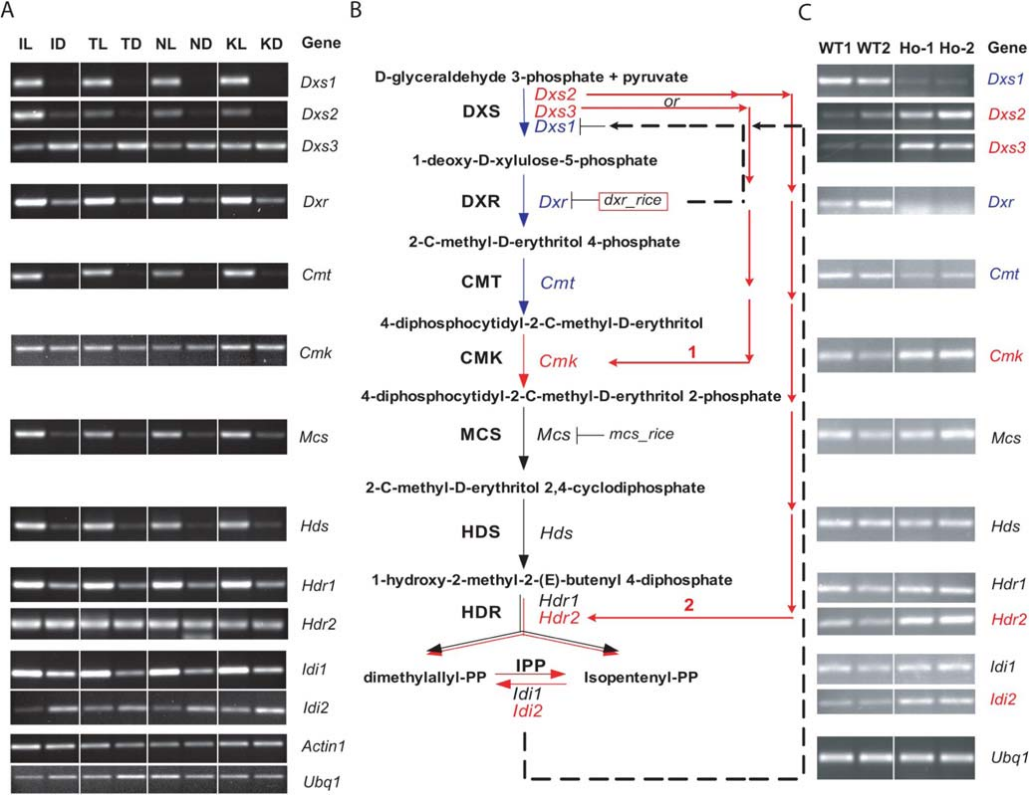
Altered Expression Patterns of Genes Associated with the MEP Pathway in the Rice *dxr* Mutant. (A) Validation of microarray data corresponding to genes in this pathway using RT-PCR amplifications. *Ubq1* and *Actin1* were used as controls for RT-PCR [85]. Numbers of PCR cycles are indicated. See legend for Figure 1 for explanation of column heading abbreviations. *Dxs1*, *Dxs2*, *Dxs3:* gene family members encoding 1-deoxy-D-xylulose-5-phosphatesynthase; *Dxr*: gene encoding 1-deoxy-D-xylulose 5-phosphate reductoisomerase (2 in B); *Cmt*: gene encoding 2-C-methyl-D-erythritol4-phosphate cytidylyl transferase (3 in B); *Cmk*: gene encoding 4-diphosphocytidyl-2-C-methyl-D-erythritol kinase (4 in B); *Mcs*: gene encoding 2-C-methyl-D-erythritol 2,4-cyclodiphosphate synthase (5 in B); *Hds*: gene encoding 4-hydroxy-3-methylbut-2-en-1-yl diphosphate synthase (6 in B); *Hdr1*, *Hdr2*: gene family members encoding 4-hydroxy-3-methylbut-2-enyl diphosphate reductase (7 in B) and there were two gene family members; and *Idi1*, *Idi2*: gene family members encoding isopentenyl-diphosphate delta-isomerase (8 in B) and there were two gene family members. (B) Proposed pathway model for explaining altered expression patterns. The MEP pathway was generated using RiceCyc (http://www.gramene.org/pathway/). Twelve candidate genes were assigned to 8 steps in the pathway. Blue colored letters and arrows indicate the suppression of gene expression in *dxr* mutant; black ones indicate not significantly changed gene expression patterns in *dxr* mutant; and red ones indicate significantly induced gene expression patterns in *dxr* mutant. Phenotypes in T-DNA insertional homozygous progenies in *Mcs* gene is displayed in Figure S8. (C) RT-PCR analysis of twelve candidate genes in this pathway in the rice *dxr* mutant (homozygous siblings) and its wild-type segregants. Genes indicated in red were up-regulated in the *dxr* mutant. Genes indicated in blue were down-regulated in the *dxr* mutant. Expression of genes indicated in black was not significantly changed in the *dxr* mutant. WT1, wild-type segregant 1; WT2, wild-type segregant 2; Ho-1, *dxr* homozygote 1; Ho-2, *dxr* homozygote 2. *Ubq1* were used as controls for RT-PCR. RT-PCR amplifications were carried out as described in Material and Methods.

In Arabidopsis, isopentenyl-PP or dimethylallyl-PP cause feedback regulation of step 1 (1-deoxy-D-xylulose-5-phosphatesynthase, DXS) of the MEP pathway (Figure 6) [72]. The co-regulation of genes controlling step 1 and step 8 suggest the existence of feedback regulation between these two steps in rice as reported in Arabidopsis [72] (Figure 4, Figure 6, and Figure S7). In an effort to deduce the hierarchical sequence of the enzymes involved in this pathway, we assessed the expression patterns of 12 genes controlling all steps in the MEP pathway in the homozygous mutant *dxr* (step2; progenies of Line 1A-14224, Ho-1 and Ho-2 in Figure 6) and its wild-type segregants (WT1 and WT2 in Figure 6). This analysis revealed that a defect at step 2 inhibits the previous step (step 1) in this pathway. These results indicate that the gene products controlling step2 and step8 of the MEP pathway cause feedback regulation of step 1 and that these three steps might be controlled by the same regulatory molecule (Figure 4, Figure 6, and Figure S7).

In addition to the predicted feedback regulation of step 2 to step 1, transcription analysis of 12 candidate genes in MEP pathway in the *dxr* mutant revealed probable compensating rotes that could be taken to make up for the defect in the family member, *Dxs1*, that is predominantly expressed in the light. There are three gene family members (*Dxs1*, *Dxs2*, and *Dxs3*) associated with step 1 (DXS). The induction in the *dxr* mutant of the two other gene family members of step1, *Dxs2* and *Dxs3*, supports a compensating roles for gene family members. The observed expression patterns of genes in other steps in this mutant facilitated predictions as to how these compensating routes would affect hierarchical sequence of the enzymes involved in this pathway. For example, as predicted, the *Dxr* (*Os01g01710*) gene at step 2 was not expressed in the mutant. The *C-methyl-D-erythritol4-phosphate cytidylyl transferase* (*Cmt,Os01g66360*) gene at step 3 was also repressed and the effect of a mutation in the *Dxr* gene might be extended to step 3 (Figure 6). However, the *Cmk* (*Os01g58790*) gene at step 4 (2-C-methyl-D-erythritol4-phosphate cytidylyl transferase 1, Cmk) was up-regulated in the rice *dxr* mutants suggesting that a compensating route might be associated with this step as well. The predominantly light-induced gene family members in the four steps following the Cmt1 (Step 4), 2-*C-methyl-D-erythritol 2,4-cyclodiphosphate synthase* (*Mcs*, *Os02g45660*), *4-hydroxy-3-methylbut-2-en-1-yl diphosphate synthase* (*Hds*, *Os02g39160*), *4-hydroxy-3-methylbut-2-enyl diphosphate reductase 1* (*Hdr1*, *Os03g52170*), and *Isopentenyl-diphosphate delta-isomerase 1* (*Idi1*, *Os07g36190*), exhibited expression levels similar to those observed for the wild-type segregants. Interestingly, *Hdr2* and *Idi2* (in step 7 and step 8 of Figure 6, respectively), which are not the predominantly expressed family members in light-grown wild-type rice, were up-regulated in the *dxr* mutants (Figure 6). This result suggested that there might be another compensation mechanism occurring to cope with the upstream blockage of the MEP pathway. As a consequence of up-regulation in step 7 (*Hdr2*, *Os03g52180*), the gene expression of step 8 (*Idi2*, *Os05g34180*) would be increased. However, these compensating routes must not to be predominant because the original route has evolved to be the most effective. Finally, plants homozygous for this mutant locus were lethal despite the functioning of these two putative compensating pathways. The specifics of these proposed compensating routes await validation through further analyses.

### Roles of the MEP Pathway in the Light Response

Most of genes involved in the carotenoid, abscisic acid, chlorophyll, and the tocopherol (Vitamin E) biosynthesis pathways, predicted to be downstream of the MEP pathway [60],[75], are light-responsive in various light *vs.* dark experiments (Figure 4 and Figure S7). These results indicate a probable metabolic connections between the MEP pathway and these four downstream pathways. The connections are likely made through intermediates synthesized via the MEP pathway in the light.

In support of the importance of this pathway in metabolism, we observed an albino leaf phenotype in homozygous progenies of lines 1A-14224 and 1C-07431, carrying T-DNA insertions in the *Dxr* gene at step 2 and 2-*C-methyl-D-erythritol 2,4-cyclodiphosphate synthase* (*Mcs*, *Os02g45660*) at step 5, respectively (Figure 3, Figure 6, and Figure S8). A mutation in Arabidopsis *Mcs1* revealed a similar albino phenotype. These plants died at the early seedling stage [76]. In Arabidopsis or tobacco, lines carrying mutations in other genes of this pathway displayed albino- or chlorotic-leaf phenotypes [72],[77],[78],[79],[80],[81]. Thus, our results indicate that the MEP pathway in rice performs key roles in generating pigments such as chlorophyll or carotenoid as it does in other plant species [31],[67],[82].

### Conclusion and Perspective

This study provides a method for identifying sets of candidate genes involved in specific biochemical pathways. Our results reveal that light regulated gene expression controls diverse metabolic networks [83],[84] and that co-expression analysis is an effective strategy for elucidating the plant response to light.

## Materials and Methods

### Sample Collection

Nipponbare, Kitaake, TP309, and IR24 rice seeds were germinated and grown in the greenhouse. Nipponbare, Kitaake, and TP309 are japonica cultivars and IR24 is indica. For light treatments seedlings remained in the greenhouse for two weeks. For dark treatments, seedlings were moved after 7 days to a dark incubator (Percival Scientific, Inc., Perry, IA) and maintained at 28°C for another 7 days.

Of 365 candidate genes showing at least an 8-fold light induction during our NSF45K light/dark experiment, 161 had T-DNA insertions in them corresponding to mutant lines in the plant functional genomics lab (PFG) and 45 of those had at least two insertionally mutated alleles (and corresponding mutant lines) present in the database (RiceGE, http://signal.salk.edu/cgi-bin/RiceGE). However, 8 of the 45 had the same or nearly the same insertion sites in the two different mutant lines and so those lines were excluded. Finally, we ordered seventy-four T-DNA insertional lines containing mutants in 37 genes. Sixty-eight of these knockout lines (japonica cv. Dongjin or Hwayoung), after excluding six lines which had insufficient seeds, were grown in the greenhouse. We observed the phenotypes of these knockout lines for 4 weeks after they germinated. The progenies showing phenotypic changes and their wild-type siblings from individual lines were harvested to extract genomic DNAs (described below) for co-segregation analyses as indicated in Figure S9. Usually, 15–20 rice plants are used for testing co-segregations and repeat two or three times this experiment. We selected lines having at least two co-segregating mutants and also repeated it at least twice.

### Genotyping of T-DNA Insertional Mutants Showing Defective Phenotypes Related to the Light Response

We visually picked out progenies displaying expected phenotypes such as color defects (albino or pale green), growth retardation or oxidative stress-related symptoms. Next, genomic DNA was extracted from mutants and from their phenotypically normal siblings (Figure S9). The genotypes of the siblings in each mutant family were determined by carrying out PCRs using two sets of primers: one designed to identify the rice gene that had been knocked out by using primers containing target gene sequences in front of and behind the T-DNA insertion site, the other designed to verify the insertion of T-DNA in the gene by using primers that amplify the *hygromycin phosphotransferase* (*hph*) gene contained within the T-DNA insert (Figure S9). The primers located upstream and downstream of the T-DNA insertional sites in each line were designed based on sequence information available from the Rice Functional Genomic Express Database (Rice GE, http://signal.salk.edu/cgi-bin/RiceGE). The PCR amplifications were carried out in 20 µl volumes of a mixture that contained 20 ng of plant DNA, 10× Taq buffer, 0.2 mM dNTP, 0.5 unit Taq polymerase (Invitrogen), and 0.2 µM of the primers for 35 cycles at 94°C for 60 s, 60°C for 60 s, and 72°C for 150 s. All primers (Sigma) for genotyping are described in Table S5.

### RNA Isolation and Reverse Transcriptase-PCR Reaction

Leaves from rice plants grown for 2 weeks in the greenhouse or in a dark incubator were collected and total RNA was isolated using TRIZOL reagent according to the manufacturer's instructions (Invitrogen, Carlsbad, CA). The total RNA was DNaseI-treated for 15 minutes then purified using the RNeasy Midi Kit (Qiagen, Germantown, MD). The total RNA was then enriched for poly-A RNA by using the Oligotex mRNA Kit (Qiagen). All steps were performed according to the manufacturer's instructions. The quantity of total RNA and mRNA were determined by measuring absorbance at 260 nm and 280 nm by using a Nanodrop ND-1000 (Nanodrop, Wilmington, DE). In addition, the level of protein contamination in the RNA was determined based on the A260/A280 ratio. Only RNA samples with ratios of 2.0–2.2 were used for these experiments. Reverse transcriptase- (RT-) PCR were carried out as used in previous study [85].

### Generation and Analysis of Microarray Data

All hybridizations were done at the Arraycore Microarray Facility at the University of California, Davis Arraycoreucdavis.edu. Probe labeling, hybridizations with the NSF45K microarray, slide scanning and identification of spot intensity were as described (Jung et al. submitted). To minimize variations caused by experimental procedures, replicated data was normalized using the Lowess normalization method in the LMGene Package [86],[87]. To identify differentially expressed genes, we used the publicly available R program LMGene developed by Rocke [86]. FDR (false discovery rate, adjusted p-value) and log_2_ fold changes of light over dark were generated for all genes. The expression data from these experiments are available through Gene Expression Ominibus (GEO) (Accession # GSE8261). To identify genes consistently expressed in response to light among different array platforms, we selected genes that were induced in our NSF45K array experiments and also showed at least 0.5 log_2_ values (1.4-fold induction) in more than two light intensity conditions of the BGI/Yale light *vs.* dark array data.

### Rice Multi-Platform Microarray Search and Analysis

The Rice Multi-platform Search page (http://www.ricearray.org/matrix.search.shtml) is a tool that allows users the ability to search across four different rice oligo microarray platform types (Affymetrix, Agilent, BGI/Yale, and NSF45K) to determine which oligos from each platform represent to a common gene target. More detailed information on Rice Multi-Platform Search Tool is available at http://www.ricearray.org/matrix.search.shtml.

The data from the BGI/Yale light *vs.* dark array dataset and the Affymetrix array data for seedling leaves and shoots for the seventeen genes in the chlorophyll biosynthesis pathway, twelve genes in the MEP pathway, and the thirty-seven genes on which functional analyses were conducted were extracted by using the rice multi-platform microarray search tool and publicly available Affymetrix and BGI/Yale array data. The detailed information on the multiplatform array data used in this study is presented in Table S6 and is also available at the NCBI GEO (http://www.ncbi.nlm.nih.gov/geo/). To make images using the multi-platform microarray data, we used TIGR MultiExperiment Viewer software (MeV, http://www.tm4.org/mev.html). We generated two tab-delimited multiple-samples files (tdms files) consisting of log_2_ ratios of light over dark treatment from the NSF45K and the BGI/Yale light *vs.* dark array datasets and of log_2_-transformed spot intensities of 23 Affymetrix array datasets related to development (Table S6). The tdms files were loaded onto the MeV and the resulting image data were used for creating Figure 2, Figure 4, Figure S6, and Figure S10.

### Analyses of Gene Families in Rice and Arabidopsis

Gene families within the predicted rice proteome were identified using Pfam and BLASTP and more detail on these identifications can be found at http://www.tigr.org/tdb/e2k1/osa1/para.family/para.method.shtml. A total of 3842 paralogous protein families containing a total of 20729 proteins were identified (http://rice.tigr.org/tdb/e2k1/osa1/para.family/index.shtml). Table S7 shows the gene families we identified in rice. To identify Arabidopsis gene family members for Figure S6, we used GenomeNet browser (http://www.genome.jp/) and the protein sequences which had more than 200 score bits or 40% similarity were considered members of the same gene family. As a result, 9 genes were identified as being unique and 23 other genes had a total of 87 additional gene family members.

### Identification of 72 Genes for Hierarchical Clustering Analysis

RiceCyc (http://pathway.gramene.org/RICE/class-instances?object=Pathways) is a web-based tool curated by Gramene (http://www.gramene.org/) and provides biochemical/metabolic pathways. Seven pathways in RiceCyc are associated with 7 mutants identified in this study. U11 is a part of the ammonia assimilation pathway and two genes in this pathway are displayed. U1 is a part of the MEP pathway and eight genes in this pathway are displayed. U10 is a part of the carotenoid biosynthesis pathway and eleven genes in this pathway are displayed. P9-1 is a part of the nitrate assimilation pathway and one gene in this pathway is displayed. P5-1 is a part of the photorespiration pathway and eight genes in this pathway are displayed. U3 is a part of the photosynthetic light reaction pathway by PSI and PSII and fifteen genes in this pathway are displayed. P13-1 is a part of the Vitamin C biosynthesis pathway and eight genes in this pathway are displayed (Figure 4; Table S4). The abscisic acid (ABA; there are 3 genes), chlorophyll (9 genes), and vitamin E biosynthetic (5 genes) pathways mediated by precursor or final product of carotenoid biosynthesis are added for co-expression analysis [31],[82] (Table S4). Unique sequences or predominantly expressed gene family members were selected for this analysis. Single-step reactions unlinked to these or other pathways were established for the other genes for which mutant phenotypes were identified during this study, i.e. U4 (*Os02g57030*), U5 (*Os03g04470*), and *Ca1* (P2-1, *Os1g45274*) (see, for example, Table 2).

### Pathway Analyses Incorporating Gene Expression Profiling Data

The pathways used in this study were developed using Gramene RiceCyc (http://pathway.gramene.org/RICE/class-instances?object=Pathways). Candidate genes in the pathways having evidence of expression, such as expressed sequence tags (ESTs) or full length cDNAs, were available at the above website. Additional candidate genes for which no evidence of gene expression was available were selected based on Arabidopsis best-hit genes homologous to TIGR version 5 gene models. Probable gene family members of all candidate genes were checked against Table S7 which lists all rice gene family members. For the co-expression analysis, we selected 72 genes in 13 biochemical/metabolic pathways or reactions associated with the 10 genes for which we had identified mutant phenotypes in this study (Figure 4). As a result, 10 gene clusters were identified. By using Cytoscape software, we deduced relationships among the different biochemical pathways or reactions (Figure S7). To do this, we considered the 10 gene clusters classified by co-expression analysis in Figure 4 as different functional groups. (The twelve boxes with different colors indicate the different biochemical pathways or reactions in Figure S7. Of them, the gray-colored box indicates reactions of unknown genes U4 and U5). In addition, co-expressed gene-clusters in Figure 4 were marked with 10 differently colored lines in Figure S7. Each line indicates an enzyme reaction carried out by the product of one of 72 genes in Figure 4. These gene products are numbered in the 10 biochemical pathways with different colored rectangular boxes (Figure S7). Each rectangular box (node) indicates chemical compounds in the pathway.

## Supporting Information

Figure S1 Previously Published Defective Phenotypes Associated with Unique Genes or Gene Family Members Predominantly Expressed in the Light involved in the Rice Chlorophyll Biosynthesis Pathway. Mutations in genes *ChlH*, *ChlI*, *ChlD*, *ChlM* and *Cao1* in the rice chloroplast biosynthesis pathway showed leaf-color defective phenotypes. White box indicates normalized spot intensity in the light and black box indicates normalized spot intensity in the dark. Numeric numbers in X-axis indicate normalized spot intensity from NSF45K array. Arrows indicate predominantly light-induced gene family members (i.e. *ChlM1* and *Cao1*). *ChlH*, magnesium-chelatase subunit H family protein gene at step 1; *ChlI*, magnesium-chelatase subunit I family protein gene at step 1; *ChlD*, magnesium chelatase ATPase subunit D protein gene at step 1; *ChlM1* and *ChlM2*, magnesium-protoporphyrin O-methyltransferase genes at step 2; and *Cao1* and *Cao2*, chlorophyll a oxygenase genes at step 7.
(1.38 MB EPS)

Figure S2 Strategy for Functional Validation of Candidate Genes.
(0.97 MB JPG)

Figure S3 Expression Patterns of 37 Rice Candidate Genes Selected For Functional Validation Based on the NSF45K Light vs. Dark Dataset. (A) Differential gene expression patterns for 12 unique genes. (B) Differential gene expression patterns for 13 gene families which include one member predominantly expressed in the light. (C) Differential gene expression patterns for 12 gene families without one predominantly expressed gene family member in the light. Arrows indicate genes for which the homozygous mutant resulted in a defective phenotype. Triangles indicate genes for which somaclonal variations mask the phenotypes in progenies homozygous for an insertional mutation in the gene. Genes which have only asterisks in (B) and sharps in (C), when homozygous for an insertional mutation, also did not segregate with a defective phenotype.
(0.48 MB JPG)

Figure S4 Examples of Mutant Lines for which Phenotypes were Not Determined Due to Apparent Somaclonal Variations. Red arrows indicate homozygous progenies. WT indicates segregants showing normal growth phenotypes. U indicates unique genes; GF, existence of gene family; P, predominantly light-induced gene family members; and NP, not predominantly light-induced gene family members. These phenotypes were repeatedly observed at least two times.
(0.42 MB JPG)

Figure S5 Phenotype Associated with the Mutation in A Not Predominantly Light-induced Gene (*Os07g05000*, *NP8-2*). Line 3A-03008 has a T-DNA insertion in rice *oxidoreductase* gene and the T-DNA insertional homozygous lines showed pale green phenotype. A black arrow indicates a homozygous progeny.
(0.09 MB JPG)

Figure S6 Arabidopsis Microarray Data of Wild Type and *pif3* Mutants Treated with Dark and Red Light for 32 Genes Which were Screened for Identifying Mutants in Photomorphogenic Signaling Pathway and their Gene Family Members. Arabidopsis microarray data of wild type and *pif3* mutants treated with dark and 1 hour red light are used for this analysis [88]. Left panel shows average log_2_ fold changes of red light over dark in wild type, red light over dark in *pif3* mutant, wild type over *pif3* mutant in dark, and wild type over *pif3* mutant in dark. Middle in this figure displays average spot intensity of dark in wild type and *pif3* mutant, and red light in wild type and *pif3* mutant. Gene_id in right panel indicates Arabidopsis gene name; numbers in the section of gene/gene family are the same with the order of 32 genes; U in the section of type indicates unique genes, P in the section of types of genes indicates predominantly light-induced gene family members, NP in the section of type indicates not predominantly light-induced gene family members, and NA indicates “not analyzed”; photomorphogenic in the section of severity of phenotype is a targeted phenotype related to phytochrome signaling pathway. Data of the phenotypes for 32 genes in this figure came from Khanna et al. [3].
(0.66 MB JPG)

Figure S7 Representation of Co-expressed Gene Clusters in Figure 4 using Cytoscape Software. Co-expression analysis of 72 genes in 13 biochemical/metabolic pathways or reactions related to mutants identified in this study identifies 10 gene clusters in Figure 4. Circles indicate steps in pathways identified by functional analyses in rice. Ten colored lines indicate 10 gene clusters in Figure 4. Eleven colored boxes indicate 11 different pathways used in this figure. Weak gray boxes indicate reactions for two unknown genes. Weak gray lines indicate the steps not analyzed for co-expression analysis. Blue box indicates methyl-erythritol phosphate (MEP); purple box, Nitrate assimilation pathway; yellow box, the reaction by carbonic anhydrase; pink box, ABA biosynthesis pathway; dark gray box, Photorespiration pathway; sky blue, Vitamin C biosynthesis pathway; red box, Carotenoid biosynthesis pathway; orange box, Ammonia assimilation pathway; green box, Chlorophyll biosynthesis pathway; lime box, Vitamin E biosynthesis pathway; white box, Photosynthetic light reaction pathway; and weak gray box, reactions by unknown genes. Numbers in each pathway indicate the order of reactions in the pathway.
(0.38 MB JPG)

Figure S8 Phenotype of *mcs* Mutant in MEP pathway.
(0.19 MB JPG)

Figure S9 Scheme for co-segregation analysis of T-DNA insertions and observed phenotypes on a large scale. Genomic DNAs were isolated from progenies showing phenotypic variation. Amplification of the *hph* gene was used to check for T-DNA insertions. p1, forward primers, in front of the T-DNA insertion site; p2, reverse primers, behind the T-DNA insertion site; p3, forward primer for identifying the *hph* gene; p4, reverse primer for identifying the *hph* gene. Upper PCR bands were produced as a result of amplifications with p1 and p2; lower PCR bands were produced as a result of amplifications with p3 and p4.
(0.12 MB JPG)

Figure S10 Expression Analysis of Rice Candidate Genes Involved in the Chlorophyll Biosynthesis Pathway Carried Out Using Publicly Available Multiplatform Array Data. Published BGI/Yale light vs. dark microarray datasets were used to check the consistency of the gene expression patterns of candidate genes in this pathway [49] (Table S6). In addition, 23 Affymetrix microarray datasets were used to compare the gene expression levels of the genes in the pathway in many different tissues and at different developmental stages (Table S6). “Two channel array” indicate the data from NSF45K and BGI/Yale arrays; “one channel array” indicates the data from the Affymetrix array. “Step” indicates the corresponding position in the chlorophyll biosynthesis pathway marked in Figure 1. 1a, 1b, and 1c indicate the genes encoding the three subunits of magnesium chelatase, respectively. Gene family members in each step are represented as −1, −2, etc. Unique means genes without gene family members; Predominant indicates a gene family member that is predominantly expressed in the light; and Not Predominant indicates a gene family member that is not the predominantly expressed gene family member in the light. An asterisk (*) indicates the existence of consistency between the gene expression patterns derived using the NSF45K and the BGI/Yale microarrays. Data in the left panel were generated by using log2 ratios from the two two-channel arrays and data in the right panel were generated using log2 transformed signal intensities from the single-channel array data. Red color in the left panel indicates light-responsive gene expression and green indicates dark-responsive expression. Yellow color in the right panel indicates a high level of gene expression and blue, a low level of gene expression. The yellow box highlights the rice light vs. dark microarray data using the NSF45K and BGI/Yale arrays. The red box highlights gene expression data from seedling leaf, seedling shoot, and young leaf derived using the Affymetrix array.
(0.47 MB JPG)

Table S1 Complete Dataset from Light vs. Dark Experiments Carried out with the Rice NSF45K Microarray. Oligo_id is the name of the NSF 45K oligo; Locus_id is the TIGR version 5 gene model based on gene locus; FDR is adjusted p-value; log2 (Light/Dark) is log2 fold change value of average normalized spot intensity in the light over average normalized spot intensity in the dark; Avg_light intensity is average normalized spot intensity in the light; Avg_dark intensity is average normalized spot intensity in the dark; std_light is standard variation in the normalized intensity in the light of all replicates and std_dark is variation in the normalized intensity in the dark of all replicates. The numbers of ESTs in nineteen tissues such as callus, root, leaf, seedling, sheath, phloem, shoot, stem, flower, panicle, anthers, pistil, endosperm, immature seed, seed, suspension, mixed, unknown, and whole are prepared. Sum of total ESTs is the summation of ESTs related to a corresponding oligo. Full length cDNA is the cDNA accession number available in Knowledge-based Oryza Molecular biological Encyclopedia (KOME, http://red.dna.affrc.go.jp/cDNA/). GO_id indicates the GO identifier accessible at AmiGO (http://amigo.geneontology.org/cgi-bin/amigo/go.cgisession7122b1203889484).
(8.19 MB XLS)

Table S2 Microarray Data Used for Figure 2. Gene_id is a simplified gene identifier; U indicates unique gene; P indicates predominantly light-induced gene family member; R indicates not predominantly light-induced gene family member; Redundancy of gene sequences is marked as U (Unique) or R (Not predominant); Predominant expression within a gene family is marked as P (Predominant); Knockout lines which were analyzed in this study are described and PFG indicates Plant Functional Genomics Laboratory in POSTECH, South Korea; Phenotypes related to light response are described; Co-segregation of observed phenotypes and T-DNA insertion is indicated as Yes or NO; Oligo_id is the name of the NSF 45K oligo; locus_id is the TIGR version 5 gene models based on gene locus; annotation indicates the putative function of the gene; NSF45K_light_vs_dark is the average log_2_ (natural light/dark) value derived using the NSF45K array data; BGI far-red_light_vs_dark is the average log_2_ (far-red light/dark) value derived using the BGI/Yale array data; BGI white_light_vs_dark is the average log_2_ (whole seedling treated with white light/dark) value derived using the BGI/Yale array data; BGI root white light_vs_dark is the average log_2_ (root treated with white light/dark) value derived using the BGI/Yale array data; BGI shoot white light_vs_dark is the average log_2_ (shoot treated with white light/dark) value derived using the BGI/Yale array data; BGI red_light_vs_dark is the average log_2_ (red light/dark) value derived using the BGI/Yale array data; *udt1-1*_anther vs WT is the average log_2_ (*udt1*-1 anther/ wild type anther) value derived using BGI/Yale array; BGI anther_meiosis_vs PL is the average log_2_ (wild type anther in meiosis/palea-lemma) value derived using BGI/Yale array; BGI_anther_young_microspore_vs PL is the average log_2_ (wild type anther in young microspore stage/palea-lemma) value derived using BGI/Yale array; BGI_anther_vacuolated_pollen_vs PL is the average log_2_ (wild type anther in vacuolated pollen stage/palea-lemma) value derived using BGI/Yale array; and BGI anther_pollen_mitosis_vs PL is the average log_2_ (wild type anther in pollen mitosis stage/palea-lemma) value derived using BGI/Yale array. The detailed information on the array data utilized is in Table S6.
(0.07 MB XLS)

Table S3 Summary of Screen for Phenotypes Associated with 17 Candidate Genes Which Were Not the Predominantly Expressed Gene Family Members in the Light or Did Not Show Consistent Gene Expression Patterns among the Microarray Datasets.
(0.08 MB DOC)

Table S4 Microarray Data Displayed in Figure 4.
(0.05 MB XLS)

Table S5 Primers used for Genotyping 20 Light-Inducible Primary Candidate Genes.
(0.07 MB DOC)

Table S6 Summary of the Rice Multiplatform Microarray Data from NCBI GEO Used for this Study.
(0.19 MB DOC)

Table S7 List of Rice Gene Family Members.
(1.86 MB XLS)

Text S1 Identification of the Primarily Light-Induced Genes among those Encoding Seven Components in the Rice Chlorophyll Biosynthesis Pathway.
(0.12 MB DOC)
