# Supplementary figures and images for: Identification and Functional Analysis of Light-Responsive Unique Genes and Gene Family Members in Rice
Source: PLoS Genet. 2008 Aug 22;4(8):e1000164. doi: 10.1371/journal.pgen.1000164 (PMC2515340; doi:10.1371/journal.pgen.1000164)

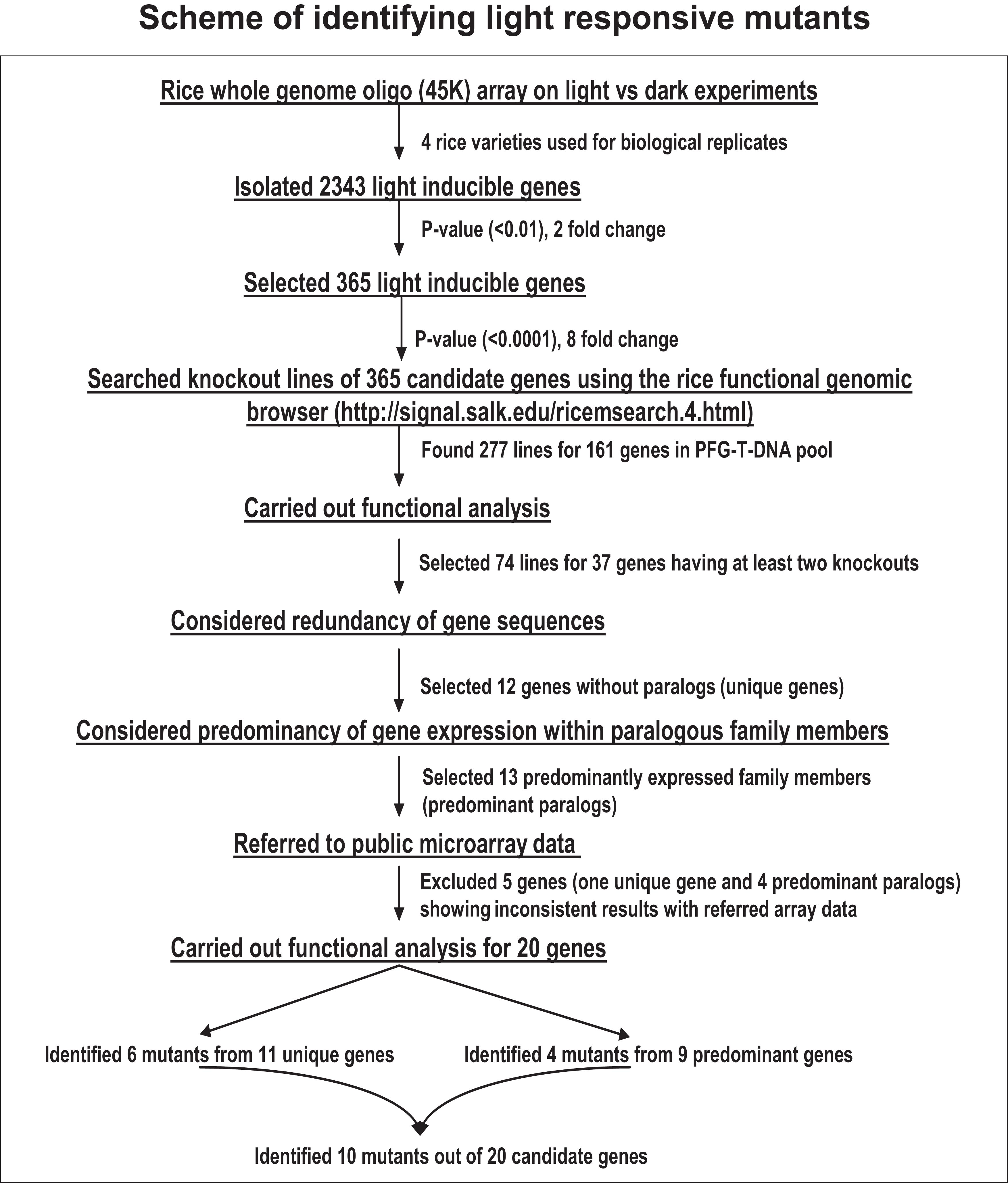

Supplement: Figure S2 — Strategy for Functional Validation of Candidate Genes. (0.97 MB JPG) [file pgen.1000164.s002.jpg]

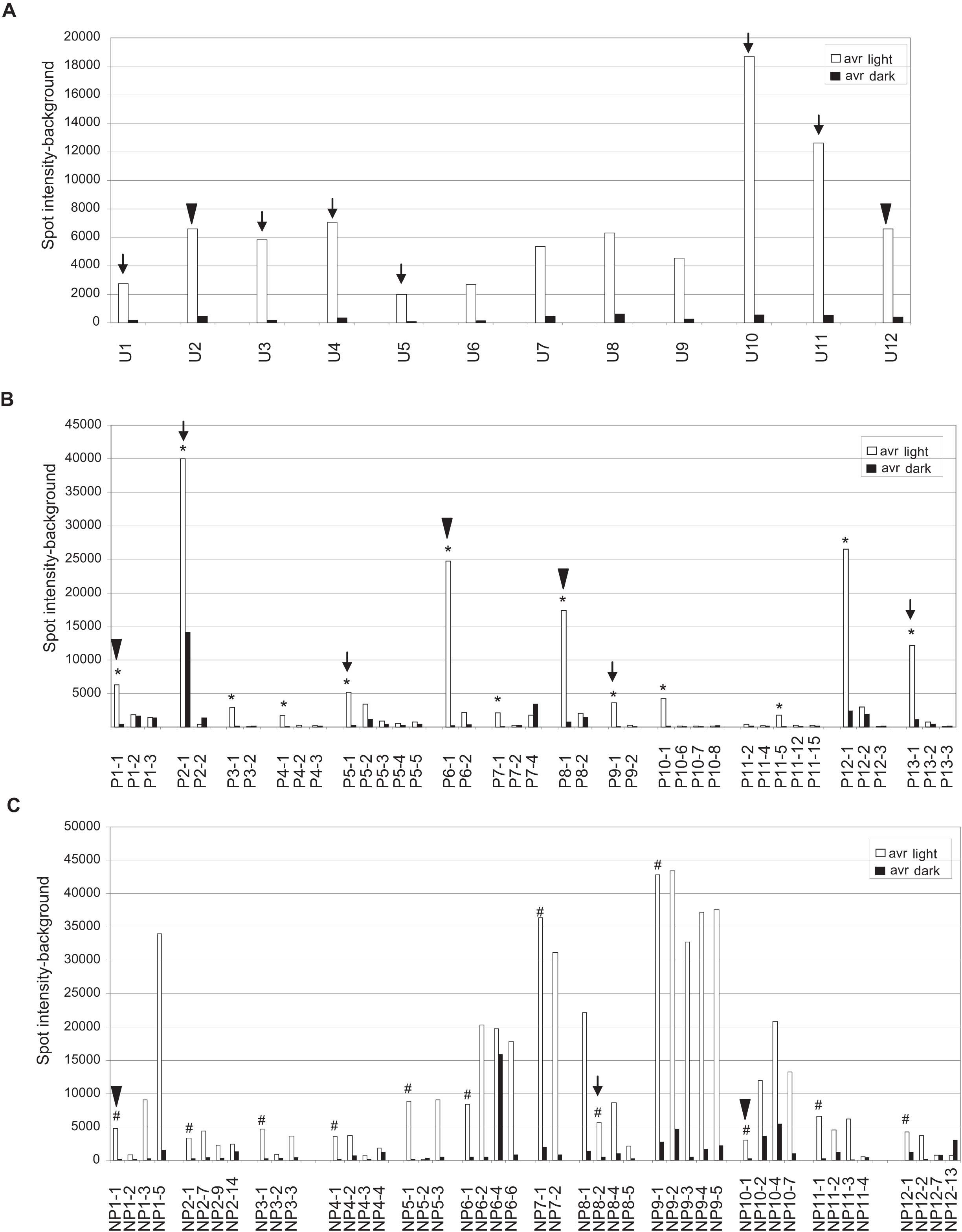

Supplement: Figure S3 — Expression Patterns of 37 Rice Candidate Genes Selected For Functional Validation Based on the NSF45K Light vs. Dark Dataset. (A) Differential gene expression patterns for 12 unique genes. (B) Differential gene expression patterns for 13 gene families which include one member predominantly expressed in the light. (C) Differential gene expression patterns for 12 gene families without one predominantly expressed gene family member in the light. Arrows indicate genes for which the homozygous mutant resulted in a defective phenotype. Triangles indicate genes for which somaclonal variations mask the phenotypes in progenies homozygous for an insertional mutation in the gene. Genes which have only asterisks in (B) and sharps in (C), when homozygous for an insertional mutation, also did not segregate with a defective phenotype. (0.48 MB JPG) [file pgen.1000164.s003.jpg]

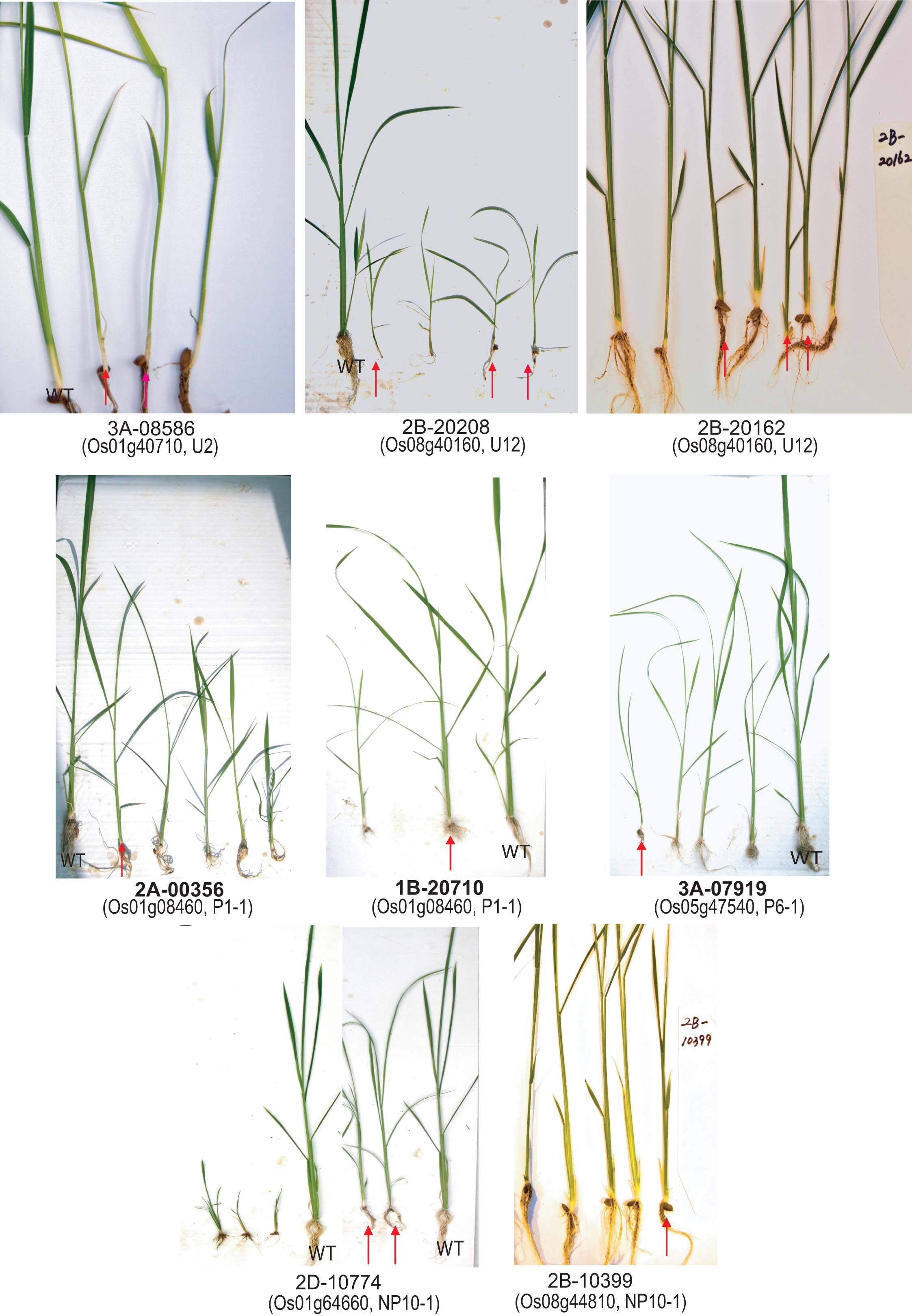

Supplement: Figure S4 — Examples of Mutant Lines for which Phenotypes were Not Determined Due to Apparent Somaclonal Variations. Red arrows indicate homozygous progenies. WT indicates segregants showing normal growth phenotypes. U indicates unique genes; GF, existence of gene family; P, predominantly light-induced gene family members; and NP, not predominantly light-induced gene family members. These phenotypes were repeatedly observed at least two times. (0.42 MB JPG) [file pgen.1000164.s004.jpg]

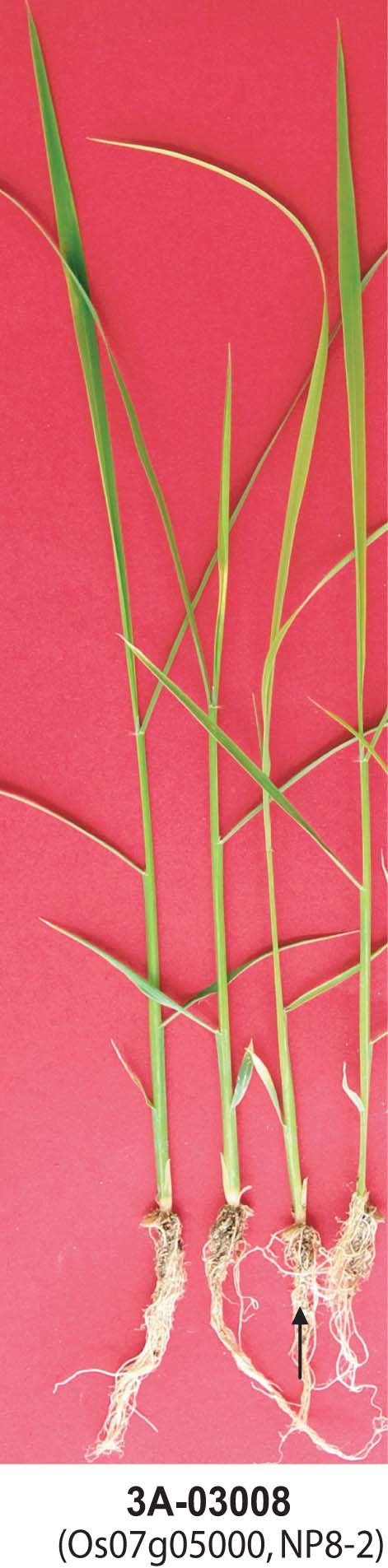

Supplement: Figure S5 — Phenotype Associated with the Mutation in A Not Predominantly Light-induced Gene (Os07g05000, NP8-2). Line 3A-03008 has a T-DNA insertion in rice oxidoreductase gene and the T-DNA insertional homozygous lines showed pale green phenotype. A black arrow indicates a homozygous progeny. (0.09 MB JPG) [file pgen.1000164.s005.jpg]

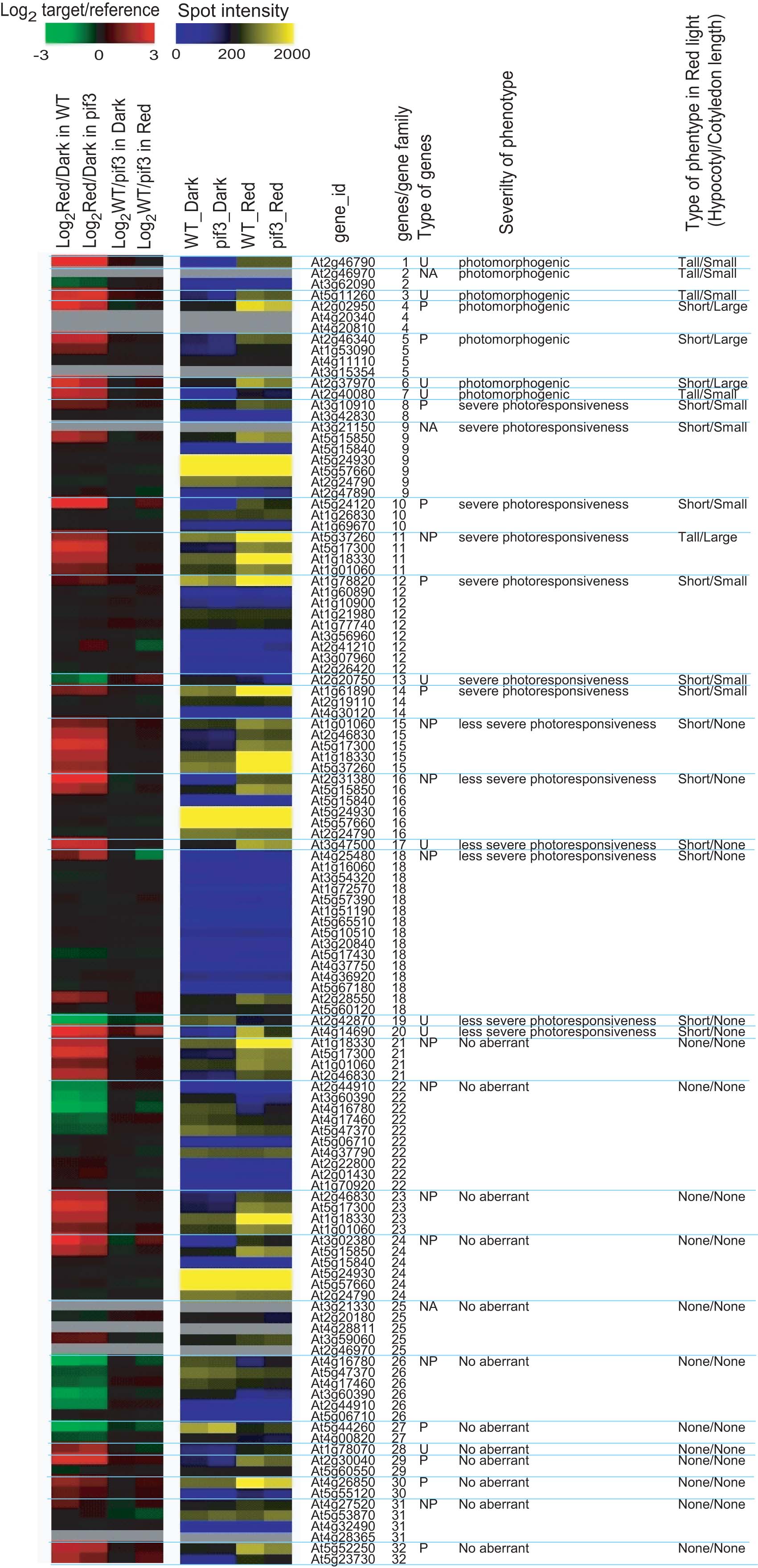

Supplement: Figure S6 — Arabidopsis Microarray Data of Wild Type and pif3 Mutants Treated with Dark and Red Light for 32 Genes Which were Screened for Identifying Mutants in Photomorphogenic Signaling Pathway and their Gene Family Members. Arabidopsis microarray data of wild type and pif3 mutants treated with dark and 1 hour red light are used for this analysis [88]. Left panel shows average log2 fold changes of red light over dark in wild type, red light over dark in pif3 mutant, wild type over pif3 mutant in dark, and wild type over pif3 mutant in dark. Middle in this figure displays average spot intensity of dark in wild type and pif3 mutant, and red light in wild type and pif3 mutant. Gene_id in right panel indicates Arabidopsis gene name; numbers in the section of gene/gene family are the same with the order of 32 genes; U in the section of type indicates unique genes, P in the section of types of genes indicates predominantly light-induced gene family members, NP in the section of type indicates not predominantly light-induced gene family members, and NA indicates “not analyzed”; photomorphogenic in the section of severity of phenotype is a targeted phenotype related to phytochrome signaling pathway. Data of the phenotypes for 32 genes in this figure came from Khanna et al. [3]. (0.66 MB JPG) [file pgen.1000164.s006.jpg]

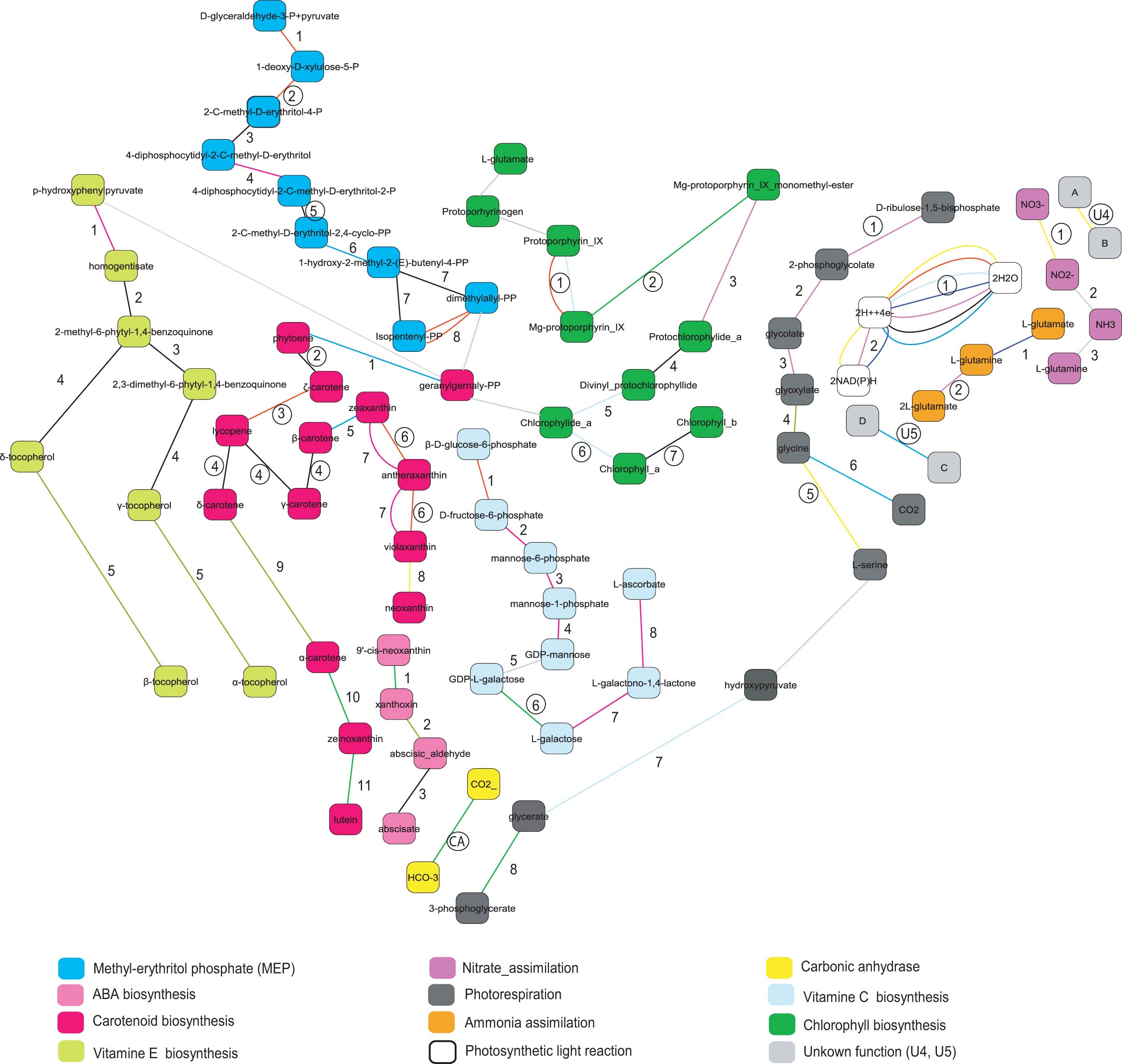

Supplement: Figure S7 — Representation of Co-expressed Gene Clusters in Figure 4 using Cytoscape Software. Co-expression analysis of 72 genes in 13 biochemical/metabolic pathways or reactions related to mutants identified in this study identifies 10 gene clusters in Figure 4. Circles indicate steps in pathways identified by functional analyses in rice. Ten colored lines indicate 10 gene clusters in Figure 4. Eleven colored boxes indicate 11 different pathways used in this figure. Weak gray boxes indicate reactions for two unknown genes. Weak gray lines indicate the steps not analyzed for co-expression analysis. Blue box indicates methyl-erythritol phosphate (MEP); purple box, Nitrate assimilation pathway; yellow box, the reaction by carbonic anhydrase; pink box, ABA biosynthesis pathway; dark gray box, Photorespiration pathway; sky blue, Vitamin C biosynthesis pathway; red box, Carotenoid biosynthesis pathway; orange box, Ammonia assimilation pathway; green box, Chlorophyll biosynthesis pathway; lime box, Vitamin E biosynthesis pathway; white box, Photosynthetic light reaction pathway; and weak gray box, reactions by unknown genes. Numbers in each pathway indicate the order of reactions in the pathway. (0.38 MB JPG) [file pgen.1000164.s007.jpg]

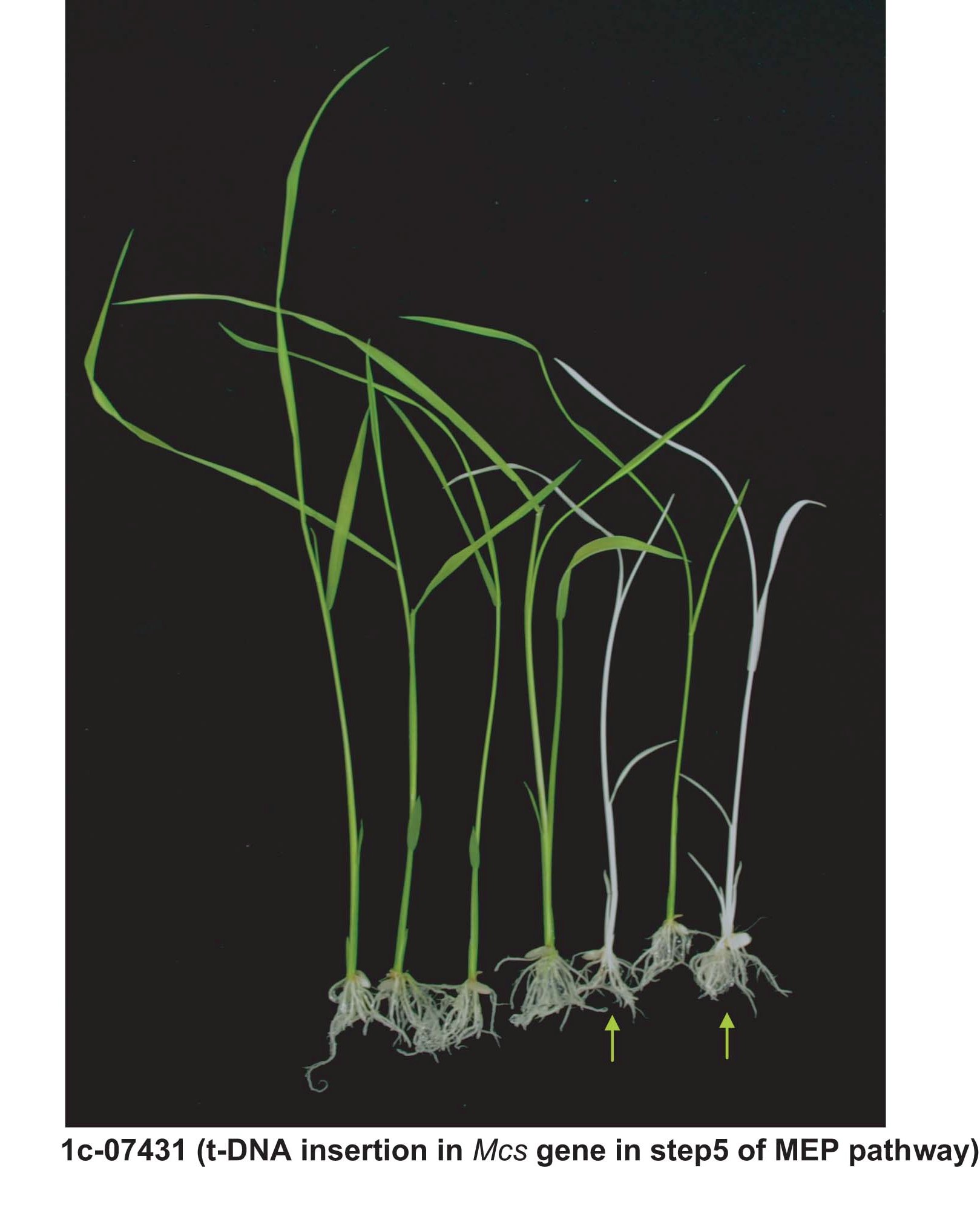

Supplement: Figure S8 — Phenotype of mcs Mutant in MEP pathway. (0.19 MB JPG) [file pgen.1000164.s008.jpg]

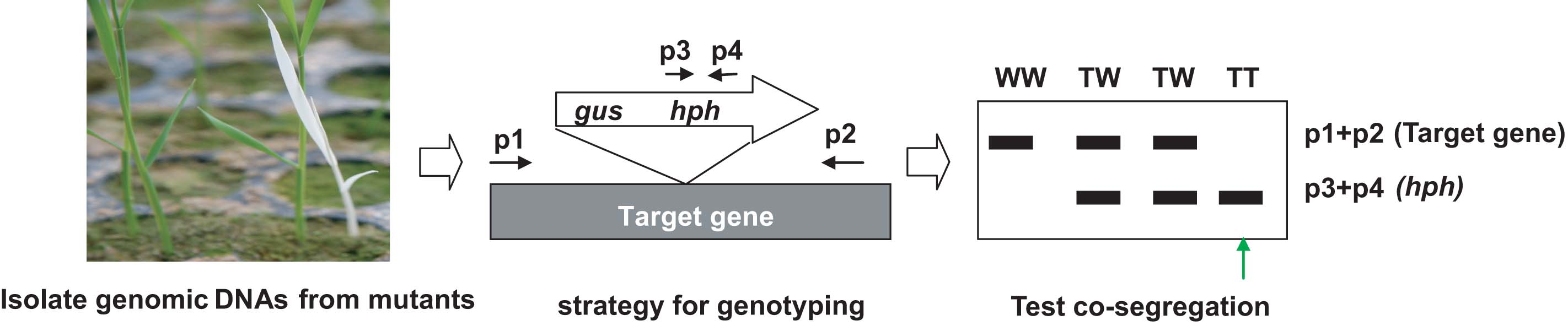

Supplement: Figure S9 — Scheme for co-segregation analysis of T-DNA insertions and observed phenotypes on a large scale. Genomic DNAs were isolated from progenies showing phenotypic variation. Amplification of the hph gene was used to check for T-DNA insertions. p1, forward primers, in front of the T-DNA insertion site; p2, reverse primers, behind the T-DNA insertion site; p3, forward primer for identifying the hph gene; p4, reverse primer for identifying the hph gene. Upper PCR bands were produced as a result of amplifications with p1 and p2; lower PCR bands were produced as a result of amplifications with p3 and p4. (0.12 MB JPG) [file pgen.1000164.s009.jpg]

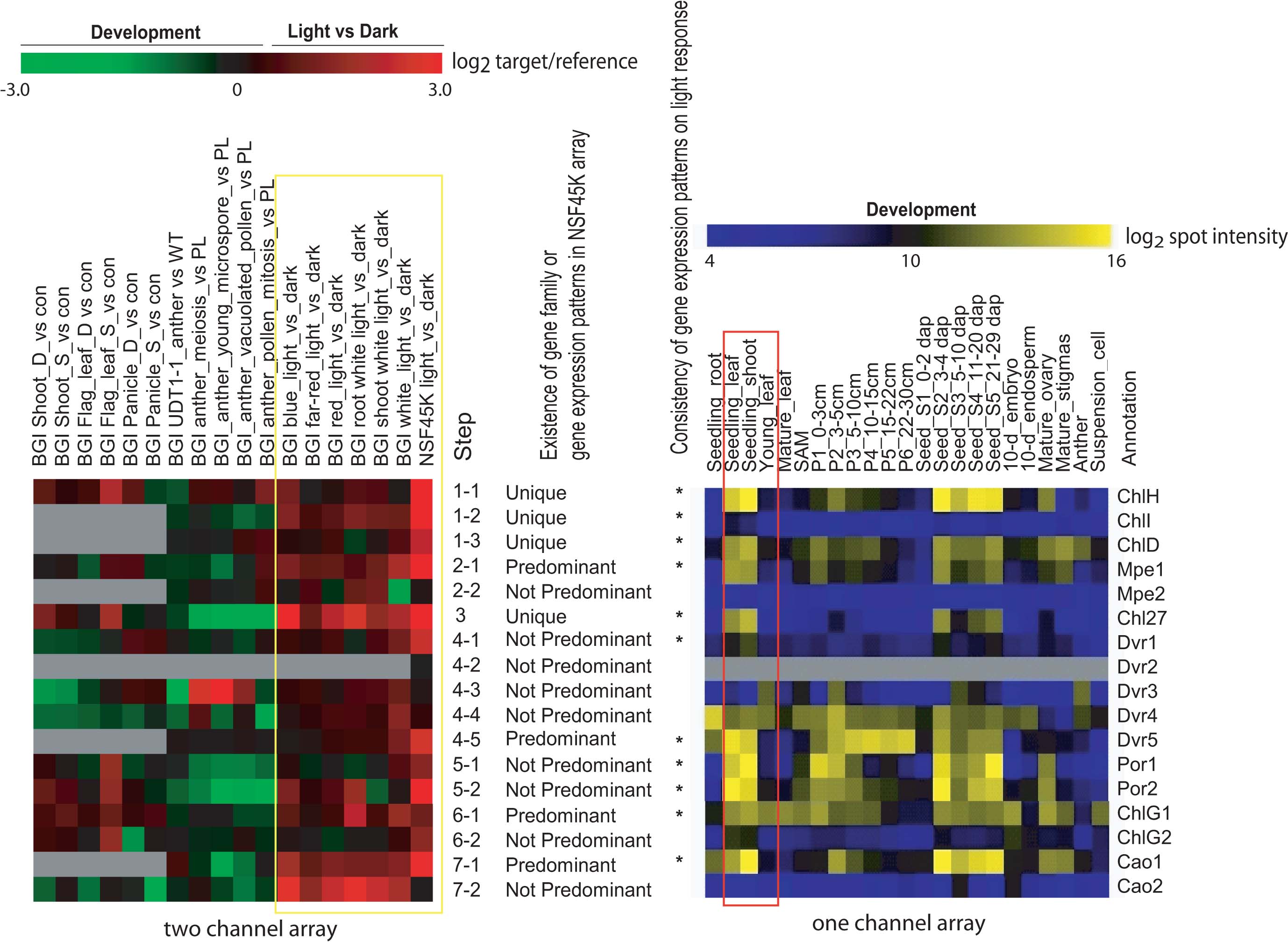

Supplement: Figure S10 — Expression Analysis of Rice Candidate Genes Involved in the Chlorophyll Biosynthesis Pathway Carried Out Using Publicly Available Multiplatform Array Data. Published BGI/Yale light vs. dark microarray datasets were used to check the consistency of the gene expression patterns of candidate genes in this pathway [49] (Table S6). In addition, 23 Affymetrix microarray datasets were used to compare the gene expression levels of the genes in the pathway in many different tissues and at different developmental stages (Table S6). “Two channel array” indicate the data from NSF45K and BGI/Yale arrays; “one channel array” indicates the data from the Affymetrix array. “Step” indicates the corresponding position in the chlorophyll biosynthesis pathway marked in Figure 1. 1a, 1b, and 1c indicate the genes encoding the three subunits of magnesium chelatase, respectively. Gene family members in each step are represented as −1, −2, etc. Unique means genes without gene family members; Predominant indicates a gene family member that is predominantly expressed in the light; and Not Predominant indicates a gene family member that is not the predominantly expressed gene family member in the light. An asterisk (*) indicates the existence of consistency between the gene expression patterns derived using the NSF45K and the BGI/Yale microarrays. Data in the left panel were generated by using log2 ratios from the two two-channel arrays and data in the right panel were generated using log2 transformed signal intensities from the single-channel array data. Red color in the left panel indicates light-responsive gene expression and green indicates dark-responsive expression. Yellow color in the right panel indicates a high level of gene expression and blue, a low level of gene expression. The yellow box highlights the rice light vs. dark microarray data using the NSF45K and BGI/Yale arrays. The red box highlights gene expression data from seedling leaf, seedling shoot, and young leaf derived usin [file pgen.1000164.s010.jpg]
