# Supplementary material for: Identification and Functional Analysis of Light-Responsive Unique Genes and Gene Family Members in Rice
Source: PLoS Genet. 2008 Aug 22;4(8):e1000164. doi: 10.1371/journal.pgen.1000164 (PMC2515340; doi:10.1371/journal.pgen.1000164)
Supplement: Table S3 — Summary of Screen for Phenotypes Associated with 17 Candidate Genes Which Were Not the Predominantly Expressed Gene Family Members in the Light or Did Not Show Consistent Gene Expression Patterns among the Microarray Datasets. (0.08 MB DOC) [file pgen.1000164.s013.doc]

**Table S3**. Summary of Screen for Phenotypes Associated with 17 Candidate Genes Which Were not Predominantly Expressed Gene Family Members or Did not Show Consistent Gene Expression Patterns.

| **T-DNA** | | | | | ***Tos17*** |
| --- | --- | --- | --- | --- | --- |
| **Locus_ID^a^**  **(Gene ID** ^b^**)** | **Annotation** | **Line^c^** | **phenotype** | **Co^d^** | **Line^c^/ phenotype** |
| **Genes showing not consistent gene expression patterns of unique genes or predominantly expressed gene family members screened** | | | | | |
| Os03g48030  (U9) | HPP family protein | 1B-22213 | dwarf | X |  |
|  |  | 2B-50151 | pale green | X |  |
| Os02g58790  (P3-1) | Expressed protein | 2A-50168 | albino | X |  |
|  |  | 2D-11229 | dwarf | X |  |
| Os09g16950  (P10-1) | lectin protein kinase | 1A-14913 | albino | X |  |
|  |  | 1A-20507 | pale green | X |  |
| Os09g37620  (P11-5) | Flavin-binding monooxygenase | 2B-10378 | pale green | X |  |
|  |  | 3A-08362 | dwarf | X |  |
| Os11g05050  (P12-1) | Stem-specific protein TSJT1 | 3D-00041 | dwarf | X |  |
|  |  | 1A-14109 | semi-dwarf | X |  |
| **Not predominantly expressed gene family members screened** | | | | | |
| Os01g64660  (NP1) | Fructose-1,6-bisphosphatase | 2C-50009 | albino | X | NC1467/ pale green |
|  |  | 2D-10774 | dwarf | ND |  |
| Os02g13560  (NP2) | Sugar transporter | 1C-06207 | dwarf | X |  |
|  |  | 2C-40140 |  | X |  |
| Os02g57160  (NP3) | ABC1 family protein | 1B-04624 | albino | X | NC0492/ No description |
|  |  | 2D-10836 | dwarf | ND |  |
| Os03g62780  (NP4) | S1 RNA binding domain protein | 1B-18127 |  | X |  |
|  |  | 3A-09602 | NA | No homo |  |
| Os04g54790  (NP5) | ABC1 family protein | 3A-10541 | slender | X |  |
|  |  | 3A-16046 | dwarf | X |  |
| Os04g57930  (NP6) | Thioredoxin X | 3D-01814 |  | X |  |
|  |  | 4A-01149 |  | X |  |
| Os06g40940  (NP7) | Gycine dehydrogenase | 2D-30154 | dwarf | X | NC6437/ pale green |
|  |  | 3D-00202 | NA | No homo |  |
| Os07g37240  (NP8) | Chlorophyll A-B binding protein | 3A-15664 | dwarf | X |  |
|  |  | 4A-00909 |  | X |  |
| Os07g05000  (NP9) | Oxidoreductase | 3A-03008 | pale green | O | ND3006/semi-dwarf |
|  |  | 3A-40003 | pale green | ND |  |
| Os08g44810  (NP10) | Malate dehydrogenase 1, chloroplast precursor | 2A-20731 | dwarf | ND |  |
|  |  | 2B-10399 | dwarf | ND |  |
| Os10g11140  (NP11) | phosphoglucomutase | 2B-20052 | NA | No homo |  |
|  |  | 3A-15337 | NA | LS |  |
| Os11g03390  (NP12) | FHA | 3A-03555 | variegated leaf | X |  |
|  |  | 2A-40278 | NA | LS |  |

^a^ TIGR Locus identifiers.

^b^ Gene identifiers used in Figure 2, Figure 3, Figure S3, Figure S4 and Figure S5.

^c^ Name of line with T-DNA or *Tos17* insertion in the candidate gene. (In most cases, two independent lines, and therefore two independent alleles, were selected for characterization.)

^d^ Results of tests for co-segregation of insertion and phenotype. O, a co-segregating line; X, a non co-segregating line; ND, co-segregation not determined due to issues involving somaclonal variation or problems with designed primers; No homo, there were no homozygous progenies; and LS, less seeds due to the very low fertility. NA, not analyzed because homozygous progenies were not available for screening or seeds were not available due to the very low fertility.
