# Supplementary material for: Identification and Functional Analysis of Light-Responsive Unique Genes and Gene Family Members in Rice
Source: PLoS Genet. 2008 Aug 22;4(8):e1000164. doi: 10.1371/journal.pgen.1000164 (PMC2515340; doi:10.1371/journal.pgen.1000164)
Supplement: Table S5 — Primers used for Genotyping 20 Light-Inducible Primary Candidate Genes. (0.07 MB DOC) [file pgen.1000164.s015.doc]

**Table S5. Primers for Genotyping of 20 Refined Candidate Genes.**

| Line | Locus Name | 5’-3’(forward) | 5’-3’(reverse) |
| --- | --- | --- | --- |
| 1C-03301 | LOC_Os01g01710 | atccatcgacgggtatttca | ggaagagctcatgccaaaac |
| 1B-14224 | LOC_Os01g01710 | tcgtgcgtacttgtcccata | ataatgcaggctgggttcac |
| 3A-08586 | LOC_Os01g40710 | tgaattccactcgcatacca | tccctactgcaaacccaaac |
| 4A-00716 | LOC_Os01g71190 | gccttggatgctcagaaaat | cttgcaagcatttatggatga |
| 2A-50128 | LOC_Os02g57030 | gaaaatcccctctgcattga | ttgcgactaatttgcgagac |
| 4A-50119 | LOC_Os02g57030 | agatggctgacacatcgaca | acaccccatacgacacccta |
| 1A-09123 | LOC_Os03g04470 | ctgatgctcatcaccgctaa | gcgaggtacgtaaggcagtt |
| 3A-03564 | LOC_Os03g04470 | ggtcaccaccaaccatttgt | ggcatagccatcaacacctt |
| 1B-01520 | LOC_Os03g06230 | gcaaagcgaggaagattttg | cctccctctttatccggttc |
| 4A-00253 | LOC_Os03g06230 | cgatgtttctttctgcgtca | attgtgctgtctgccctacc |
| 1B-01342 | LOC_Os03g19760 | tgctgtgtggaagtgaatttg | acacatgcatcaagggatca |
| 1C-06809 | LOC_Os03g19760 | attccaatgtcagcgactcc | tgttccgacgagaacctacc |
| 1A-17021 | LOC_Os03g47610 | tgcatttgtcgtcatcttcag | cgtgtgagttccccttgagt |
| 1A-08723 | LOC_Os03g47610 | ttccctctctttccccctaa | caaggtgctcctttggtgtt |
| 3A-16669 | LOC_Os04g37619 | gtgctggagagggacatgag | tccagacttcgcactctcct |
| 4A-50669 | LOC_Os04g37619 | gcctaattaatccgtcattagca | aggcttgatagacgcaacca |
| 1D-02443 | LOC_Os07g46460 | cgtccagatgcaacctgtaa | tgttgatctcgccttctgtg |
| 3A-01082 | LOC_Os07g46460 | aaaggcctgacgacgtagaa | gggttttgaaaagctcgatg |
| 2B-20162 | LOC_Os08g40160 | tcagtcagcgtagccatcac | ttacgcttgctctgatggaa |
| 2B-20208 | LOC_Os08g40160 | agaaaagaacacgccctttg | cgatttccaccttccaaaaa |
| 2B-00356 | LOC_Os01g08460 | gcaggaaaactcaattcatcc | tttgctttccacattccaca |
| 1B-20710 | LOC_Os01g08460 | aattgcgcggctagatattg | atcaaagccgctgatttctg |
| 2B-60065 | LOC_Os01g45274 | ccagccagctggagttttac | tcgataaggaggcagcagat |
| 3C-00294 | LOC_Os01g45274 | aggaatatgcctgtggttgc | ggatcaaacgatcggctcta |
| 2C-10006 | LOC_Os03g37830 | tttcacatggggtatttgtatttg | tagctagctgcccggttaaa |
| 2D-40669 | LOC_Os03g37830 | ttcgtgagtttatggcgttt | ttgagacctcctgcaacaga |
| 3A-07919 | LOC_Os05g47540 | gctgtcatgtgggctatacg | tcggagagggttatgtcagc |
| 1D-03944 | LOC_Os03g52840 | ggataggggcaaaaaggttc | cccaccagagacaagttcgt |
| 2A-10285 | LOC_Os07g32880 | gaggatctggctgttgaggt | tctgccatgaagttttgtgc |
| 3D-01688 | LOC_Os07g32880 | acggcgatatccctcctaat | ctactgcaggatgccaggat |
| 3A-02083 | LOC_Os06g04510 | aaacgccgcctttttaagat | atggcaagccataggaggta |
| 3A-12320 | LOC_Os06g04510 | catggtccataggcacaaga | ggtggtattggttccgttca |
| 2D-10946 | LOC_Os08g36480 | ctcgatcgaacaaatgctca | tgcgccttcaatttcttttt |
| 4A-50280 | LOC_Os08g36480 | cagaacatggtgcagcagac | tcgccaccagaatggatatt |
| 3A-14221 | LOC_Os12g08810 | aagatggtgagcgaagagga | cctcagaggattgaccagga |
| 3A-50953 | LOC_Os12g08810 | cccctgtccaataggaatga | cagcgttcccattatgtgtg |
| All lines | *hph* | gatgttggcgacctcgtatt | gcgaagaatctcgtgctttc |
