# Supplementary material for: Identification and Functional Analysis of Light-Responsive Unique Genes and Gene Family Members in Rice
Source: PLoS Genet. 2008 Aug 22;4(8):e1000164. doi: 10.1371/journal.pgen.1000164 (PMC2515340; doi:10.1371/journal.pgen.1000164)
Supplement: Table S6 — Summary of the Rice Multiplatform Microarray Data from NCBI GEO Used for this Study. (0.19 MB DOC) [file pgen.1000164.s016.doc]

**Table S6**. Summary of Rice Multiplatform Microarray Data in NCBI GEO Used for This Study.

| Platform | Experiments | Number of replicates | GEO accession # | Reference |
| --- | --- | --- | --- | --- |
| Affymetrix | Seedling leaf | 3 | GSE6901 | [1] |
| Affymetrix | Seedling root | 1 | GSE7951 | [1] |
| Affymetrix | Seedling shoot | 1 | GSE7951 | [1] |
| Affymetrix | Shoot apical meristem (SAM) | 3 | [GSE6893](http://www.ncbi.nlm.nih.gov/geo/query/acc.cgi?acc=GSE6893) | [2] |
| Affymetrix | Young leaf | 3 | [GSE6893](http://www.ncbi.nlm.nih.gov/geo/query/acc.cgi?acc=GSE6893) | [2] |
| Affymetrix | Mature leaf | 3 | [GSE6893](http://www.ncbi.nlm.nih.gov/geo/query/acc.cgi?acc=GSE6893) | [2] |
| Affymetrix | P1 (0-3 cm panicle, immature panicle) vs | 3 | [GSE6893](http://www.ncbi.nlm.nih.gov/geo/query/acc.cgi?acc=GSE6893) | [2] |
| Affymetrix | P2 (3-5 cm panicle) | 3 | [GSE6893](http://www.ncbi.nlm.nih.gov/geo/query/acc.cgi?acc=GSE6893) | [2] |
| Affymetrix | P3 (5-10 cm panicle, meiotic stage) | 3 | [GSE6893](http://www.ncbi.nlm.nih.gov/geo/query/acc.cgi?acc=GSE6893) | [2] |
| Affymetrix | P4 (10-15 cm panicle, young microspore stage) | 3 | [GSE6893](http://www.ncbi.nlm.nih.gov/geo/query/acc.cgi?acc=GSE6893) | [2] |
| Affymetrix | P5 (15-22 cm panicle, vacuolated pollen stage ) | 3 | [GSE6893](http://www.ncbi.nlm.nih.gov/geo/query/acc.cgi?acc=GSE6893) | [2] |
| Affymetrix | P6 (22-30 cm panicle, mature pollen stage) | 3 | [GSE6893](http://www.ncbi.nlm.nih.gov/geo/query/acc.cgi?acc=GSE6893) | [2] |
| Affymetrix | S1 (0-2 dap^a^, early globular embryo) | 3 | [GSE6893](http://www.ncbi.nlm.nih.gov/geo/query/acc.cgi?acc=GSE6893) | [2] |
| Affymetrix | S2 (3-4 dap^a^, middle and late globular embryo) | 3 | [GSE6893](http://www.ncbi.nlm.nih.gov/geo/query/acc.cgi?acc=GSE6893) | [2] |
| Affymetrix | S3 (5-10 dap^a^, embryo morphogenesis) | 3 | [GSE6893](http://www.ncbi.nlm.nih.gov/geo/query/acc.cgi?acc=GSE6893) | [2] |
| Affymetrix | S4 (11-20 dap^a^, embryo maturation | 3 | [GSE6893](http://www.ncbi.nlm.nih.gov/geo/query/acc.cgi?acc=GSE6893) | [2] |
| Affymetrix | S5 (21-29 dap^a^, dormancy and desiccation tolerance) | 3 | [GSE6893](http://www.ncbi.nlm.nih.gov/geo/query/acc.cgi?acc=GSE6893) | [2] |
| Affymetrix | Ovary | 3 | GSE7951 | [1] |
| Affymetrix | Stigma | 3 | GSE7951 | [1] |
| Affymetrix | Anther | 1 | GSE7951 | [1] |
| Affymetrix | 10 dap^a^ embryo | 1 | GSE7951 | [1] |
| Affymetrix | 10 dap^a^ endosperm | 1 | GSE7951 | [1] |
| Affymetrix | Suspension cell | 1 | GSE7951 | [1] |
| BGI/Yale | blue light vs dark in whole seedling | 3 | GSE2619 | [3] |
| BGI/Yale | red light vs dark in whole seedling | 3 | GSE2619 | [3] |
| BGI/Yale | far-red light vs dark in whole seedling | 3 | GSE2619 | [3] |
| BGI/Yale | white light vs dark in whole seedling | 3 | GSE2619 | [3] |
| BGI/Yale | white light vs dark in root | 3 | GSE2619 | [3] |
| BGI/Yale | white light vs dark in shoot | 3 | GSE2619 | [3] |
| BGI/Yale | Seedling vs suspension cell | 3 | GSE2691 | [4] |
| BGI/Yale | Shoot vs suspension cell | 3 | GSE2691 | [4] |
| BGI/Yale | Root vs suspension cell | 3 | GSE2691 | [4] |
| BGI/Yale | Panicle 1 (young panicle) vs cc (suspension cell culture) | 3 | GSE2691 | [4] |
| BGI/Yale | Panicle 2 (mature panicle) vs cc (suspension cell culture) | 3 | GSE2691 | [4] |
| BGI/Yale | BGI Shoot_D_vs con | 4 | GSE6533 | [5] |
| BGI/Yale | BGI Shoot_S_vs con | 3 | GSE6533 | [5] |
| BGI/Yale | BGI Flag_leaf_D vs con | 4 | GSE6533 | [5] |
| BGI/Yale | BGI Flag_leaf_S_vs con | 3 | GSE6533 | [5] |
| BGI/Yale | BGI Panicle_D_vs con | 4 | GSE6533 | [5] |
| BGI/Yale | BGI Panicle_S_vs con | 3 | GSE6533 | [5] |
| BGI/Yale | BGI *udt1-1*_anther vs WT | 3 | GSE2619 | [6] |
| BGI/Yale | BGI anther_meiosis_vs PL | 2 | GSE2619 | [6] |
| BGI/Yale | BGI_anther_young_microspore_vs PL | 2 | GSE2619 | [6] |
| BGI/Yale | BGI_anther_vacuolated_pollen_vs PL | 2 | GSE2619 | [6] |
| BGI/Yale | BGI anther_pollen_mitosis_vs PL | 2 | GSE2619 | [6] |
| NSF45K | Natural light vs dark in leaf, 4 varieties, 14 day old seedling | 8 | GSE8261 | This study |

^a^ Indicates “ day after pollination”.

D indicates drought-stress; S, salt-stress; con, untreated samples; PL, palea-lemma; and WT, wild-type.

1. Li M, Xu W, Yang W, Kong Z, Xue Y (2007) Genome-Wide Gene Expression Profiling Reveals Conserved and Novel Molecular Functions of the Stigma in Rice (Oryza sativa L.). Plant Physiol.

2. Jain M, Nijhawan A, Arora R, Agarwal P, Ray S, et al. (2007) F-box proteins in rice. Genome-wide analysis, classification, temporal and spatial gene expression during panicle and seed development, and regulation by light and abiotic stress. Plant Physiol 143: 1467-1483.

3. Jiao Y, Yang H, Ma L, Sun N, Yu H, et al. (2003) A genome-wide analysis of blue-light regulation of Arabidopsis transcription factor gene expression during seedling development. Plant Physiol 133: 1480-1493.

4. Ma L, Chen C, Liu X, Jiao Y, Su N, et al. (2005) A microarray analysis of the rice transcriptome and its comparison to Arabidopsis. Genome Res 15: 1274-1283.

5. Zhou J, Wang X, Jiao Y, Qin Y, Liu X, et al. (2007) Global genome expression analysis of rice in response to drought and high-salinity stresses in shoot, flag leaf, and panicle. Plant Mol Biol 63: 591-608.

6. Jung KH, Han MJ, Lee YS, Kim YW, Hwang I, et al. (2005) Rice Undeveloped Tapetum1 is a major regulator of early tapetum development. Plant Cell 17: 2705-2722.
