# Supplementary material for: Identification and Functional Analysis of Light-Responsive Unique Genes and Gene Family Members in Rice
Source: PLoS Genet. 2008 Aug 22;4(8):e1000164. doi: 10.1371/journal.pgen.1000164 (PMC2515340; doi:10.1371/journal.pgen.1000164)
Supplement: Text S1 — Identification of the Primarily Light-Induced Genes among those Encoding Seven Components in the Rice Chlorophyll Biosynthesis Pathway. (0.12 MB DOC) [file pgen.1000164.s018.doc]

**Supplemental Text**

**Identification of the Primarily Light-induced Genes among those Encoding Seven Components in the Rice Chlorophyll Biosynthesis Pathway**

## To assess the usefulness of our microarray data we surveyed the expression patterns we obtained through microarray analysis for seventeen genes, including gene family members, which encode proteins for the seven steps in the well-characterized chlorophyll biosynthetic pathway (Figure 1). This rice chlorophyll biosynthetic pathway was developed using RiceCyc (http://acorn.cshl.org/pathway/), which is a pathway tool for rice curated by Gramene (http://www.gramene.org/pathway/). The pathway was also somewhat modified after referring to recent reports in Arabidopsis [1,2].

It was straightforward to consider the unique genes with significantly light-responsive expression patterns among the seventeen genes as having critical roles in the pathway, but it was not as simple to identify potential targets for subsequent functional studies if there was more than one candidate gene at a step of the pathway. The expression patterns of families of genes from our microarray data gave us a way to choose the genes that were better candidates for involvement in each pathway step at which the enzyme involved was encoded by a gene that belonged to a multi-gene family. According to gene families data obtained from TIGR (<http://www.tigr.org/tdb/e2k1/osa1/para.family/para.method.shtml>; see Materials and Methods for more detail; Table S7), five of the enzymes in the chlorophyll biosynthetic pathway are encoded by genes that belong to gene families in rice. These enzymes are: magnesium-protoporphyrin O-methyltransferase (CHLM, step 2), divinyl protochlorophyllide reductase (DVR, step 4), protochlorophyllide reductase (POR, step 5), chlorophyll synthase (CHLG, step 6), and chlorophyll a oxygenase (CAO, step 7). We found, based on our light *vs*. dark NSF45K microarray data, that most of the unique genes associated with steps of this pathway in rice were induced by light and we identified the member of each of the five gene families that was the most predominantly expressed family member in the light associated with steps 2 and 4-7 as well (Figure 1).

The expression patterns of all of the candidate genes in the seven steps of this pathway were validated using reverse transcriptase- (RT-) PCR (See Materials and Methods) (Figure 1). Results indicated that most of the genes [designated 1a (Os03g20700), 1b (Os03g36540), 1c (Os03g59640), 2-1 (Os06g04150), 3 (Os01g17170), 4-1 (Os03g22780), 4-4 (Os08g34280), 4-5 (Os09g25150), 5-1 (Os04g58200), 5-2 (Os10g35370), 6-1 (Os05g28200), 6-2 (Os03g09060), and 7-1 (Os10g41780) in Figure 1] were induced in the light, results similar to those obtained using the microarray (Figure 1). On the other hand, we could not detect consistent differential changes among different genotypes between light *vs.* dark conditions in the case of gene *Os02g35060* (2-2) and that of gene *Os10g41760* (7-2)*.* The gene expression patterns were similar among the three japonica varieties (i.e. Kitaake, Nipponbare, and Tapei309) whereas gene expression patterns were different between subspecies. Reverse transcriptase- (RT-) PCR again supports that differences in genetic background clearly affect the expression patterns.

In addition, it was difficult to detect expression of gene *Os02g56690* and gene *Os08g17500* even after 35 cycles of RT-PCR (see genes 4-2 and 4-3, respectively, in Figure 1); however, these RT-PCR results are reasonably consistent with our microarray data (Figure 1). Our findings corroborate the light-inducibility of these genes as demonstrated in Arabidopsis [1]. We therefore conclude that most of the genes in this pathway are well represented by their corresponding oligos on the NSF45K microarray.

Magnesium chelatase, at step 1 is known to be comprised of multiple subunits (designated 1a, 1b and 1c in Figure 1) [1,3,4,5,6,7] but whether other enzymes in the pathway consist of multiple subunits is not clear. Nevertheless, we suspected that the enzymes at steps 4 (DVR) and 6 (CHLG) would both be redundantly or cooperatively encoded by two gene family members since two candidate genes from each family associated with these steps showed significant induction in the light (Figure 1). On the other hand, we expected that the enzymes at step 2 (CHLM), step 5 (POR), and step 7 (CAO) would be primarily generated as a result of the expression of one predominantly light-responsive member (designated 2-1, 5-2 and 7-1, respectively) of their respective gene families (Figure 1). These three genes were consequently designated the “predominantly light-induced gene family member” associated with their respective enzymes in this pathway. The three subunits of the magnesium chelatase complex carrying out the reaction at step 1, magnesium-chelatase subunit H (CHLH), magnesium-chelatase subunit I (CHLI) and magnesium-chelatase subunit D (CHLD), and the enzyme magnesium-protoporphyrin IX monomethyl ester cyclase (MPE) at step 3 are encoded by light-inducible genes that do not belong to gene families in rice (Figure 1). Therefore, these four are referred to as “unique genes”. With the possible exceptions of steps 4 (4-1 and 4-5) and 6 (6-1 and 6-2) for which two gene family members appeared similarly induced in the light, these results indicated that the other five steps in the chlorophyll biosynthesis pathway of rice were straightforward targets for reverse genetics studies of the pathway because they each appeared to be primarily controlled through the regulation of one gene.

Based on gene expression profiling data obtained using the NSF45K, we concluded that the four unique genes (1a, 1b, 1c, and 3) and the three predominantly light-responsive candidate genes (2-1, 5-2, and 7-1) would be good targets for studying the biological functions of genes involved in rice chlorophyll biosynthesis. And, as one would therefore expect, previous studies in rice showed that the knockout mutants of the three unique genes encoding the subunits comprising magnesium chelatase complexes in rice, CHLH (1a), CHLI (1b), and CHLD (1c), exhibited chlorina phenotypes [6,7] (Figure S1). The remaining unique gene at step 3 was expected to have a function similar to that of its Arabidopsis ortholog, *CHL27* [8]. Also, the functions of rice *ChlM* (*Os06g04150*; gene 2-1) and rice *Cao1* (*Os10g41780*; gene 7-1) had been previously identified by studying knockout lines of these predominantly light-induced family members [9,10] (Figure S1). Similarly, mutation in gene 5-2 (*Os10g35370*) have more probability to show phenotypic change but the phenotypes has not yet been determined. However, among genes which were not the most highly light-induced member of their respective family, the function of rice *ChlG* (*Os05g28200*; gene 6-1) had been studied previously using missense mutations [11]. No other light-related mutant phenotypes had been identified for any other member of a family of genes associated with this pathway in rice.

To help us figure out the reason why a mutation in gene 6-1 revealed phenotypes we referred to other publicly available microarray data on the responses of genes after illumination with different light intensities [12]. Figure S10 shows that gene 6-1 displayed significant light-induction in response to more light treatments than did gene 6-2. Based on these gene expression patterns, we hypothesize that the product of gene 6-1 product carries out the predominant role at this step in the pathway. Subsequently, we compared all of our NSF45K-derived results to other publicly available expression profiles of genes in rice to effectively refine our list of the most important candidate genes involved in the chlorophyll biosynthesis pathway.

## **Comparison of the NSF45K Array Data with Other Microarray Data**

We compared our light *vs.* dark microarray dataset with publicly available datasets on light *vs.* dark gene expression responses produced by a group using the Beijing Genomics Institute (BGI/Yale) microarray platform [12]. The BGI/Yale datasets were derived from 6 comparisons: whole seedlings subjected to blue light *vs.* darkness, whole seedlings subjected to far-red light *vs.* darkness, whole seedlings subjected to red light *vs.* darkness, whole seedlings subjected to white light *vs.* darkness, roots subjected to white light *vs.* darkness, and shoots subjected to white light *vs.* darkness (Figure S10). The data of Jiao et al. [12] were generated using 3 hybridizations and containing 3 biological replicates per each treatment. Other BGI/Yale array datasets that compared gene expression in samples harvested from other tissues to suspension cultured cell or in developing anthers relative to palea/lemma (i.e., outer hulls of rice spikelet) were used to see various responses of selected candidate genes besides light response [13,14] (Figure S10). In addition, we obtained an Affymetrix microarray dataset derived from experiments carried out with tissues or organs of rice harvested at various developmental stages and used the expression levels of the chlorophyll biosynthesis pathway genes measured in seedling leaves and seedling shoots to check for possible redundancy among the same gene family members in terms of gene expression level [12,15,16]. A multiplatform search tool developed by TIGR (http://www.ricearray.org/matrix.search.shtml; see Materials and Methods) allowed us to get the information on the oligos from these other array platforms (such as the BGI/Yale and Affymetrix rice arrays), which were mapped to common TIGR gene models.

The expression patterns of the seventeen candidate chlorophyll biosynthesis-related genes that were analyzed using the NSF45K array were compared to the results obtained with data from other array platforms using the multiplatform search tool. Primarily, we examined the consistency of significant light responses in the BGI/Yale and NSF45K light *vs.* dark array datasets (those marked with asterisks in Figure S10) and found that the light-inducible patterns associated with the genes designated 1a, 1b, 1c, 2-1, 3, 4-1, 4-5, 5-1, 5-2, 6-1, and 7-1 were most consistent between both platforms (i.e., NSF45K and BGI/Yale) (Figure S10). Then, expression profiles in seedlings, developing panicles and seeds, and specific tissues in mature flowers obtained using the rice Affymetrix array were used to examine the expression levels of our 17 selected candidate genes and checked against the expression levels of these genes in leaves and shoots at the seedling stage to estimate expression levels of individual gene family members within a multi-gene family during the conditions under which the light *vs* dark experiments were carried out (Figure S10). Of the analyzed candidate genes, the expression of 5-2 and 5-1 genes was similar in seedling leaves and shoots in the Affymetrix data (Figure 10). This result suggests that these two genes may function redundantly in the rice chlorophyll biosynthesis process. When we used only the NSF45K-derived light *vs.* dark data, the predominantly expressed gene family members were 2-1, 5-2, and 7-1. After applying publicly available microarray data, our list of candidate genes as reliable targets for further functional approaches can include 6-1 and exclude 5-2 (Figure S10).

**Supplemental Materials and Methods**

**Probe labeling**

Labeled probes for hybridizations with the NSF45K microarray were prepared from mRNA samples using the SuperScript^TM^ indirect cDNA Labeling System (Invitrogen). This system utilizes a secondary labeling method and thereby avoids the dye bias commonly associated with direct incorporation of dye-modified nucleotides during the reverse transcription reaction. Briefly, this procedure involves the incorporation of amino allyl-dUTP during cDNA synthesis followed by coupling of the amino allyl-modified cDNA with a fluorochrome (Cy3 or Cy5). The *in vitro* reverse transcription was performed by using 1 μg mRNA combined with random hexamers and oligo (dT) primers and incubating for 3 hours at 46°C in a final volume of 30 μl containing SuperScript^TM^ III Reverse Transcriptase (400 U/μl), 5x reaction buffer, 0.1 M DTT, and a dNTP mixture including an amino allyl-modified nucleotide (AA-dUTP). Prior to reverse transcription, the RNA template was hydrolyzed using 15 μl of 1 N NaOH (70°C, 15 min) followed by neutralization with 15 μl of 1N HCl. Unincorporated primers and nucleotides were removed using the S.N.A.P.^TM^ Column purification system according to the manufacturer’s protocol (Invitrogen) and the purified amino allyl-modified cDNA was re-suspended in 5 μl of the coupling buffer supplied by manufacturer. The amino allyl-modified cDNA was then mixed with lyophilized Cy3 or Cy5 that had been re-suspended in 5 μl dimethylsulfoxide (Sigma-Aldrich Corp., St. Louis, MO) and the mixture incubated for 1 hr at room temperature in the dark. The reaction was quenched by adding 15 μl of 4 M hydroxylamine (15 min, room temperature in the dark). The dye-coupled cDNA was then purified by using the S.N.A.P.^TM^ Column purification system.

**Microarray hybridizations and scanning**

All hybridizations were done at the Arraycore Microarray Facility at the University of California, Davis (Arraycore@ucdavis.edu). Prior to hybridizations, microarrays were treated with a solution containing sodium borohydride (NaBH_4_) to minimize non-specific autofluorescence from the spotted material. Briefly, slides were placed into a solution containing 2x SSC (0.3 M sodium chloride, 0.03 M sodium citrate, pH 7.0)/0.05% SDS/0.25% NaBH_4_ (**Rohm and Haas Company**, Philadelphia, PA) and incubated at 42°C for 20 min. Slides were transferred to 1x SSC for 5 min at room temperature and then sequentially washed with vigorous stirring using fresh 1x SSC (3 × 5 min, room temperature), 0.2x SSC (4 × 2 min, room temperature), and Nanopure (Millipore, Milford, MA) water (1 × 2 min, room temperature). Slides were spin-dried (1000 rpm, 10 min) and stored under argon until use.

Hybridizations were performed in a clean room environment (HEPA- and carbon-filtered) to minimize exposure of microarrays and labeled probes to dust and ozone. Microarray pre-hybridization, hybridization, and washes were performed using an HS4800 Automated Slide Hybridization Station (Tecan, Switzerland). Cy3- or Cy5-labeled cDNA probes were mixed and dried by vacuum centrifugation. Probes were suspended in 100 µl GeneFrames hybridization solution (MWG Biotech, UK) and incubated in boiling water for 3 min, centrifuged (14000×g, 5 min), and left at room temperature until injection into the hybridization station. Microarray slides were pre-hybridized in the hybridization station for 15 min at 50°C in 5x SSPE/6M Urea/0.5% Tween-20/10x Denhardt’s solution (Sigma). Samples were hybridized for 16 hours at 50°C with medium agitation, then sequentially washed with 2x SSC/0.2% SDS [2 × (1 min wash, 1 min soak, 37°C)], 1x SSC [2 × (1 min wash, 1 min soak, 37°C)], and 0.5x SSC [2 × (1 min wash, 1 min soak, 30°C)], and dried under N_2_ (5 min, 30°C). Slides were kept under N_2_ until they were scanned.

Hybridized microarray slides were imaged using a GenePix 4000B dual laser microarray scanner (Molecular Devices, Sunnyvale, CA) at 5 µm resolution. Slides were imaged using 100% laser power for both lasers (532 and 635 nm) and scanned twice using the high Photo Multiplier Tube (PMT) and low PMT settings.

**Microarray data processing and normalization**

Spot intensities were quantified using Axon GenePix Pro 4.0 image analysis software (http://www.fh-co.com/Axon_files/GN_GenePix_Support.html). Afterwards, GenePix Pro 4.0 result data files (.gpr files) were generated using the high PMT and low PMT settings. For high PMT, we normalized replicated data to minimize the variations caused by experimental procedures using the Lowess normalization method in the LMGene Package [17,18]. We further normalized signal intensity among different experiments using averages of all the gene signals obtained during individual experiments. In addition, we estimated background “expression” level based on the signal associated with the gene from the binary vector pCAMBIA-1305 encoding hygromycin phosphotransferase (*hph*, GenBank Accession: AF354045), included on the array as a negative control. Probes were prepared from light grown leaf and dark grown leaf of 4 different rice varieties and they were both simultaneously hybridized to a single microarray. Then, probes generated from each variety were dye-swapped. From resulting 8 hybridizations, we got an average, normalized spot intensity for the *hph* oligos, which were evenly spotted throughout the slides, of 220 (with a standard deviation of 30). We then generated average normalized spot intensities following a common strategy (Kim et al., 2003; Ma et al., 2005) by subtracting average *hph* intensity and adding 2 standard deviations of the average normalized *hph* intensity. At the adjusted average normalized spot intensity, 60 was the real background level and the threshold for expression was estimated as 90 after adding the standard deviation (30) to the adjusted background level (60). We found that 43.5% of all the genes represented on the NSF45K array had an average normalized spot intensity of at least 90. To identify differentially expressed genes, we used the publicly available R program LMGene developed by Rocke (2004). FDR (false discovery rate, adjusted p-value) and fold changes of light over dark were generated for all genes (Table S1). The expression data from these experiments are available through Gene Expression Ominibus (GEO) (Accession # GSE8261). To identify genes consistently expressed in response to light among different array platforms, we selected genes that were induced in our NSF45K array experiments and also showed at least 0.5 log_2_ values (1.4-fold induction) in more than two light intensity conditions of the BGI/Yale light *vs* dark array data.

**Supplemental References**

1. Matsumoto F, Obayashi T, Sasaki-Sekimoto Y, Ohta H, Takamiya K, et al. (2004) Gene expression profiling of the tetrapyrrole metabolic pathway in Arabidopsis with a mini-array system. Plant Physiol 135: 2379-2391.

2. Nagata N, Tanaka R, Satoh S, Tanaka A (2005) Identification of a vinyl reductase gene for chlorophyll synthesis in Arabidopsis thaliana and implications for the evolution of Prochlorococcus species. Plant Cell 17: 233-240.

3. Grafe S, Saluz HP, Grimm B, Hanel F (1999) Mg-chelatase of tobacco: the role of the subunit CHL D in the chelation step of protoporphyrin IX. Proc Natl Acad Sci U S A 96: 1941-1946.

4. Mochizuki N, Brusslan JA, Larkin R, Nagatani A, Chory J (2001) Arabidopsis genomes uncoupled 5 (GUN5) mutant reveals the involvement of Mg-chelatase H subunit in plastid-to-nucleus signal transduction. Proc Natl Acad Sci U S A 98: 2053-2058.

5. Jensen PE, Willows RD, Petersen BL, Vothknecht UC, Stummann BM, et al. (1996) Structural genes for Mg-chelatase subunits in barley: Xantha-f, -g and -h. Mol Gen Genet 250: 383-394.

6. Zhang H, Li J, Yoo JH, Yoo SC, Cho SH, et al. (2006) Rice Chlorina-1 and Chlorina-9 encode ChlD and ChlI subunits of Mg-chelatase, a key enzyme for chlorophyll synthesis and chloroplast development. Plant Mol Biol 62: 325-337.

7. Jung KH, Hur J, Ryu CH, Choi Y, Chung YY, et al. (2003) Characterization of a rice chlorophyll-deficient mutant using the T-DNA gene-trap system. Plant Cell Physiol 44: 463-472.

8. Tottey S, Block MA, Allen M, Westergren T, Albrieux C, et al. (2003) Arabidopsis CHL27, located in both envelope and thylakoid membranes, is required for the synthesis of protochlorophyllide. Proc Natl Acad Sci U S A 100: 16119-16124.

9. Lee S, Kim JH, Yoo ES, Lee CH, Hirochika H, et al. (2005) Differential regulation of chlorophyll a oxygenase genes in rice. Plant Mol Biol 57: 805-818.

10. Fujino K, Sekiguchi H, Kiguchi T (2005) Identification of an active transposon in intact rice plants. Mol Genet Genomics 273: 150-157.

11. Wu Z, Zhang X, He B, Diao L, Sheng S, et al. (2007) A chlorophyll-deficient rice mutant with impaired chlorophyllide esterification in chlorophyll biosynthesis. Plant Physiol 145: 29-40.

12. Jiao Y, Ma L, Strickland E, Deng XW (2005) Conservation and divergence of light-regulated genome expression patterns during seedling development in rice and Arabidopsis. Plant Cell 17: 3239-3256.

13. Jung KH, Han MJ, Lee YS, Kim YW, Hwang I, et al. (2005) Rice Undeveloped Tapetum1 is a major regulator of early tapetum development. Plant Cell 17: 2705-2722.

14. Ma L, Chen C, Liu X, Jiao Y, Su N, et al. (2005) A microarray analysis of the rice transcriptome and its comparison to Arabidopsis. Genome Res 15: 1274-1283.

15. Jain M, Nijhawan A, Arora R, Agarwal P, Ray S, et al. (2007) F-box proteins in rice. Genome-wide analysis, classification, temporal and spatial gene expression during panicle and seed development, and regulation by light and abiotic stress. Plant Physiol 143: 1467-1483.

16. Li M, Xu W, Yang W, Kong Z, Xue Y (2007) Genome-wide gene expression profiling reveals conserved and novel molecular functions of the stigma in rice. Plant Physiol 144: 1797-1812.

17. Rocke DM (2004) Design and analysis of experiments with high throughput biological assay data. Semin Cell Dev Biol 15: 703-713.

18. Berger JA, Hautaniemi S, Jarvinen AK, Edgren H, Mitra SK, et al. (2004) Optimized LOWESS normalization parameter selection for DNA microarray data. BMC Bioinformatics 5: 194-206.
